# Isolation and functional characterization of hepatitis B virus-specific T-cell receptors as new tools for experimental and clinical use

**DOI:** 10.1371/journal.pone.0182936

**Published:** 2017-08-08

**Authors:** Karin Wisskirchen, Kai Metzger, Sophia Schreiber, Theresa Asen, Luise Weigand, Christina Dargel, Klaus Witter, Elisa Kieback, Martin F. Sprinzl, Wolfgang Uckert, Matthias Schiemann, Dirk H. Busch, Angela M. Krackhardt, Ulrike Protzer

**Affiliations:** 1 Institute of Virology, Technische Universität München / Helmholtz Zentrum München, Munich, Germany; 2 German Centre for Infection Research (DZIF), Munich partner site, Munich, Germany; 3 III. Medical Department, Klinikum rechts der Isar, Technische Universität München, Munich, Germany; 4 Laboratory for Immunogenetics and Molecular Diagnostics, Klinikum der Universität München, Munich, Germany; 5 Institute of Biology, Humboldt-University Berlin, Max-Delbrück-Center for Molecular Medicine in the Helmholtz Association and Berlin Institute of Health, Berlin, Germany; 6 Institute for Medical Microbiology, Immunology and Hygiene, Technische Universität München, Munich, Germany; 7 Focus Groups “Viral Hepatitis” and “Clinical Cell Processing and Purification”, Institute for Advanced Study, Technische Universität München, Munich, Germany; Maisonneuve-Rosemont Hospital, CANADA

## Abstract

T-cell therapy of chronic hepatitis B is a novel approach to restore antiviral T-cell immunity and cure the infection. We aimed at identifying T-cell receptors (TCR) with high functional avidity that have the potential to be used for adoptive T-cell therapy. To this end, we cloned HLA-A*02-restricted, hepatitis B virus (HBV)-specific T cells from patients with acute or resolved HBV infection. We isolated 11 envelope- or core-specific TCRs and evaluated them in comprehensive functional analyses. T cells were genetically modified by retroviral transduction to express HBV-specific TCRs. CD8^+^ as well as CD4^+^ T cells became effector T cells recognizing even picomolar concentrations of cognate peptide. TCR-transduced T cells were polyfunctional, secreting the cytokines interferon gamma, tumor necrosis factor alpha and interleukin-2, and effectively killed hepatoma cells replicating HBV. Notably, our collection of HBV-specific TCRs recognized peptides derived from HBV genotypes A, B, C and D presented on different HLA-A*02 subtypes common in areas with high HBV prevalence. When co-cultured with HBV-infected cells, TCR-transduced T cells rapidly reduced viral markers within two days. Our unique set of HBV-specific TCRs with different affinities represents an interesting tool for elucidating mechanisms of TCR-MHC interaction and dissecting specific anti-HBV mechanisms exerted by T cells. TCRs with high functional avidity might be suited to redirect T cells for adoptive T-cell therapy of chronic hepatitis B and HBV-induced hepatocellular carcinoma.

## Introduction

Chronic hepatitis B affects 257 million people worldwide and a curative treatment does not exist [1]. A cure of HBV infection is characterized by a full control of virus replication and disappearance of circulating hepatitis B surface (HBs) antigen [2]. Adoptive T-cell therapy, in which the viral transcription template covalently closed circular DNA (cccDNA) is eliminated or controlled by the immune system, might allow HBV cure or at least functional cure.

In chronic infection T cells fail to control HBV in infected hepatocytes so that the virus can persist [3]. Adoptive T-cell therapy intends to mimic the T-cell response that is mounted during naturally resolving, acute hepatitis B [4] by infusion of HBV-specific T cells that can secrete antiviral cytokines and/or kill infected hepatocytes [5]. To this end, the patient´s T cells can be rendered HBV-specific by introduction and expression of receptors that target the infected cells [6]. These receptors can either be chimeric antigen receptors (CAR) or cloned, natural TCRs. CARs are artificial molecules with an antibody domain binding HBs protein on infected cells and intracellular TCR stimulation domains that activate the T cell. CARs function independently from major histocompatibility complex (MHC)-presentation and can theoretically be used in every patient [7,8]. On the other hand, certain pairs of natural α and β TCR chains bind an MHC-molecule loaded with an HBV peptide. They can be isolated, cloned and used to genetically engineer T cells [9,10]. A natural TCR is restricted to a distinct peptide and a distinct MHC molecule, but has the advantage of activating the T cell in its physiological way and is presumably more sensitive.

Adoptive transfer of T cells equipped with TCRs recognizing tumor antigens showed promising results in the treatment of malignant diseases [11–13]. The strength of a T-cell response and hence its antitumor or antiviral activity are defined by the avidity of the TCRs [14,15]. TCRs can not only be expressed in CD8^+^ but also in CD4^+^ T cells. This allows CD4^+^ T cells to recognize MHC-I-restricted epitopes, which likely increases the success of adoptive T-cell therapy [16,17]. In this regard, it is important that the TCR is of high affinity and thus does not depend on CD8 co-receptor binding [18].

In the present study, we aimed at generating a set of HBV-specific TCRs with high avidity to identify promising candidates that are suitable for adoptive T-cell therapy. We identified 11 TCRs specific for three different HBV peptides presented on HLA-A*02. A thorough characterization of these 11 HBV-specific TCRs allowed us to define their specificity and unravel their functional avidity in assays that take into account TCR affinity, TCR avidity and clustering, co-receptor binding and physiological peptide presentation [19]. Some of the TCRs displayed a high functional avidity in both CD8^+^ and CD4^+^ T cells and allowed T cells transduced with these TCRs to achieve a rapid antiviral activity against HBV infection.

## Results

### HBV-specific T-cell clones can be isolated from peripheral blood mononuclear cells of individuals with resolved HBV infection

Three HLA-A*02^+^ donors with already resolved or acute, resolving HBV infection were selected to clone high-affinity TCRs (Table 1). Donor 1 had anti-HBs titers of >1000 IU/ml, was HBV-DNA-negative and had resolved HBV infection around 25 years before. Donor 2 had a prolonged course of an acute hepatitis B (2.9x10^7^ IU/ml HBV-DNA), which was cleared after one year receiving entecavir treatment. Patient 3 had an acute infection with 1.9x10^6^ IU/ml HBV-DNA, which spontaneously decreased to 48.8 IU/ml ten weeks later, pointing at a resolving course of infection (Table 1).

**Table 1:** Characteristics of donors at the time of PBMC isolation.

Characteristics	Donor 1	Donor 2	Donor 3
Sex	M	F	M
Age (years)	49	52	61
HBV infection status	resolved	acute, resolving	acute, resolving
HLA-A	02:01, 23:01	01:01, 02:01	02:01
HLA-B	39:24, 44:03	08:01, 44:02	07:02, 41:02
HLA-C	04:01, 07:01	05:01, 07:01	12:03, 17:03
HLA-DRB1	07:01, 13:03	04:04, 13:01	13:03, 15:01
HLA-DQB1	02:02, 03:01	03:02, 06:03	03:01, 05:02
HLA-DPB1	02:01, 04:01	04:02, 06:01	04:01

**Fig 1:**
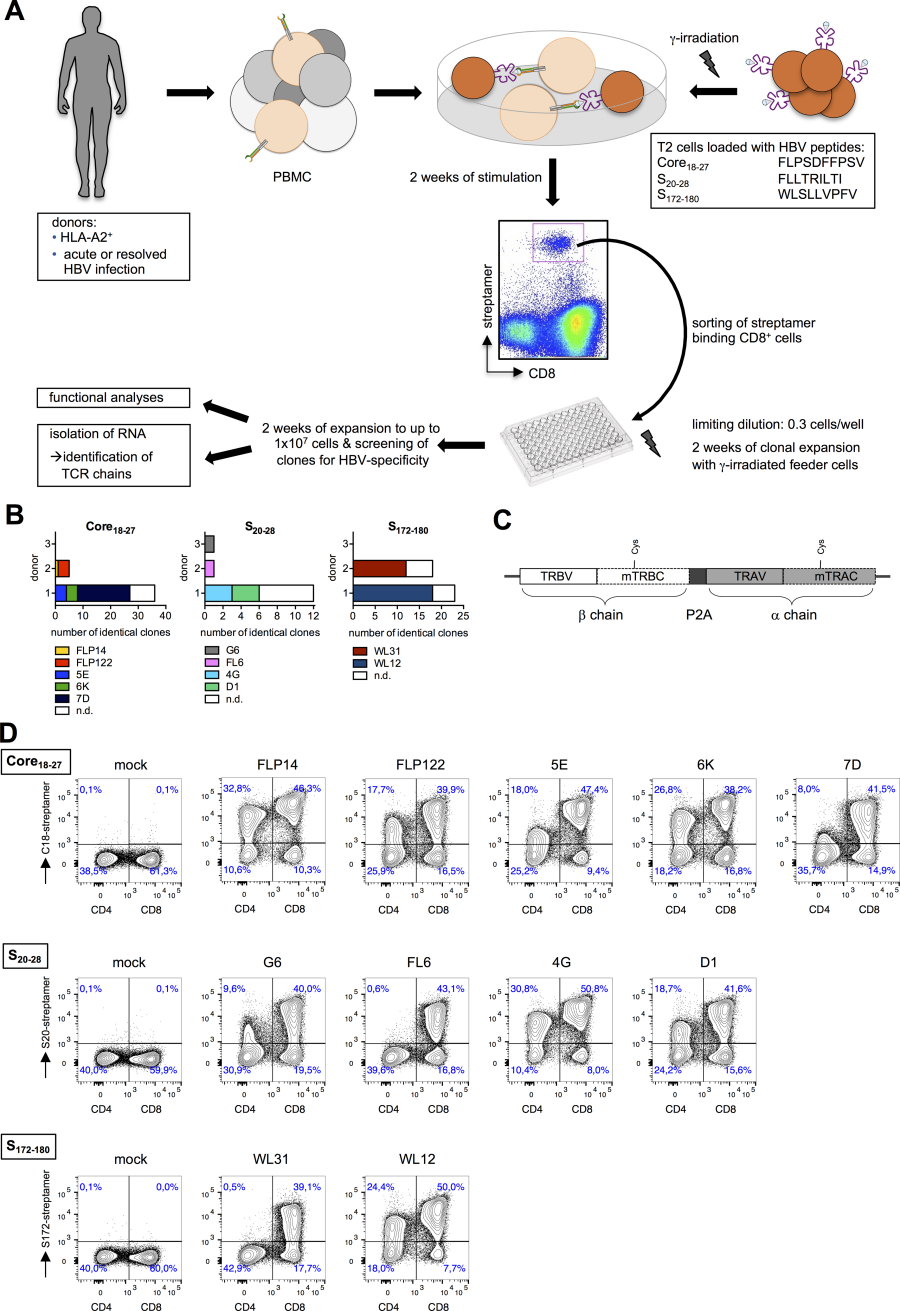
Isolation of HBV-specific T cells. (A) Experimental procedure to isolate HBV-specific T cells. T cells from donor 1 with acute, donor 2 with protracted and donor 3 with resolving HBV infection were stimulated for two weeks with peptide-loaded, irradiated TAP-deficient T2 cells or by direct addition of HBV-derived peptides to PBMC. HBV-specific CD8^+^ T cells were isolated using reversible MHC multimers (MHC Streptamers) and cloned by limiting dilution. T-cell clones were expanded and subsequently analyzed for their specificity. (B) TCR β chains of all T-cell clones with specificity for HBV peptide C18, S20 or S172 were sequenced; n.d. = not determined because of unclear sequencing results. Each color represents identical TCR sequences. (C) Schematic representation of both TCR chains cloned as one transgene cassette into the retroviral vector MP71. Gene sequences were codon-optimized, constant regions were murinized introducing an additional disulfide bond, and TCR α and β chains were fused by a P2A element for polycystronic expression. (D) TCR-expressing T cells were generated by retroviral transduction: retroviral supernatant was obtained by transient transfection of 293T cells with gene-optimized constructs and packaging plasmids derived from the murine leukemia virus. Pre-stimulated PBMC were spinoculated two times on the plates coated with retrovirus supernatant. Successfully transduced T cells were identified by CD3, CD4 or CD8 and MHC Streptamer staining.

HBV-specific T cells were expanded with HBV peptides C18 (Core_18-27_), S20 (S_20-28_) and S172 (S_172-180_) of proven immunogenicity [20]. After two weeks, cells were stained with reversible HLA-A*02 multimers (Streptamers) [21], isolated using flow cytometry-based cell sorting and clonally expanded thereafter (Fig 1A, S1 Table). In total, about 400 T-cell clones were obtained, 100 of which showed HBV-specific cytotoxicity and were subjected to PCR-based TCR sequence analyses. Sequence comparison of individual TCR chains revealed that several T-cell clones from one patient shared an identical TCR indicating clonal expansion from the same precursor cell (Fig 1B). Interestingly, for the 11 different TCRs defined, we could detect a common usage of TCR chains among different donors, i.e. Vα17 and Vβ12 recognizing peptide S20, or Vα12/13 and Vβ27 for C18 (S2 Table). Functional avidity of 11 different T-cell clones was determined by titrating the corresponding peptide in a chromium release killing assay (S1 Fig). Activation by nano- or picomolar concentration of peptide suggested that we had obtained potent HBV-specific T-cell clones, with C18-specific clones being the most sensitive ones.

We next sought to compare the functionality of the TCRs independently from the general fitness of the T-cell clone. First, complete TCR α and β chains were cloned into separate retroviral vectors and expression after retroviral transduction of peripheral blood mononuclear cells (PBMC) was confirmed by MHC multimer staining (S2 Fig). Secretion of interferon (IFN)-γ in a co-culture assay revealed that cells loaded with HBV-derived peptides specifically activated transduced T cells. We noticed that the specific response of T cells primarily correlated with the expression level of the respective TCR with e.g. G6 and D1 being expressed at low and FLP14 and FLP122 at high levels, respectively (S2 Fig). Therefore, in a next step TCR expression was improved by optimizing codon-usage, inserting an additional disulfide bond, exchanging constant regions for their murine counterparts and ensuring equimolar translation of both TCR chains from one construct using a P2A element [22] (Fig 1C and S3 Fig). By this, expression was enhanced about two-fold (S3 Fig) and up to 80% of T cells expressed the various TCRs as indicated by MHC multimer staining (Fig 1D) or by staining against the murine constant domain (S3 Fig). Thus, we generated a set of 11 functional TCRs with optimized expression recognizing different HBV proteins.

### T cells genetically engineered to express HBV-specific TCRs become polyfunctional

**Fig 2:**
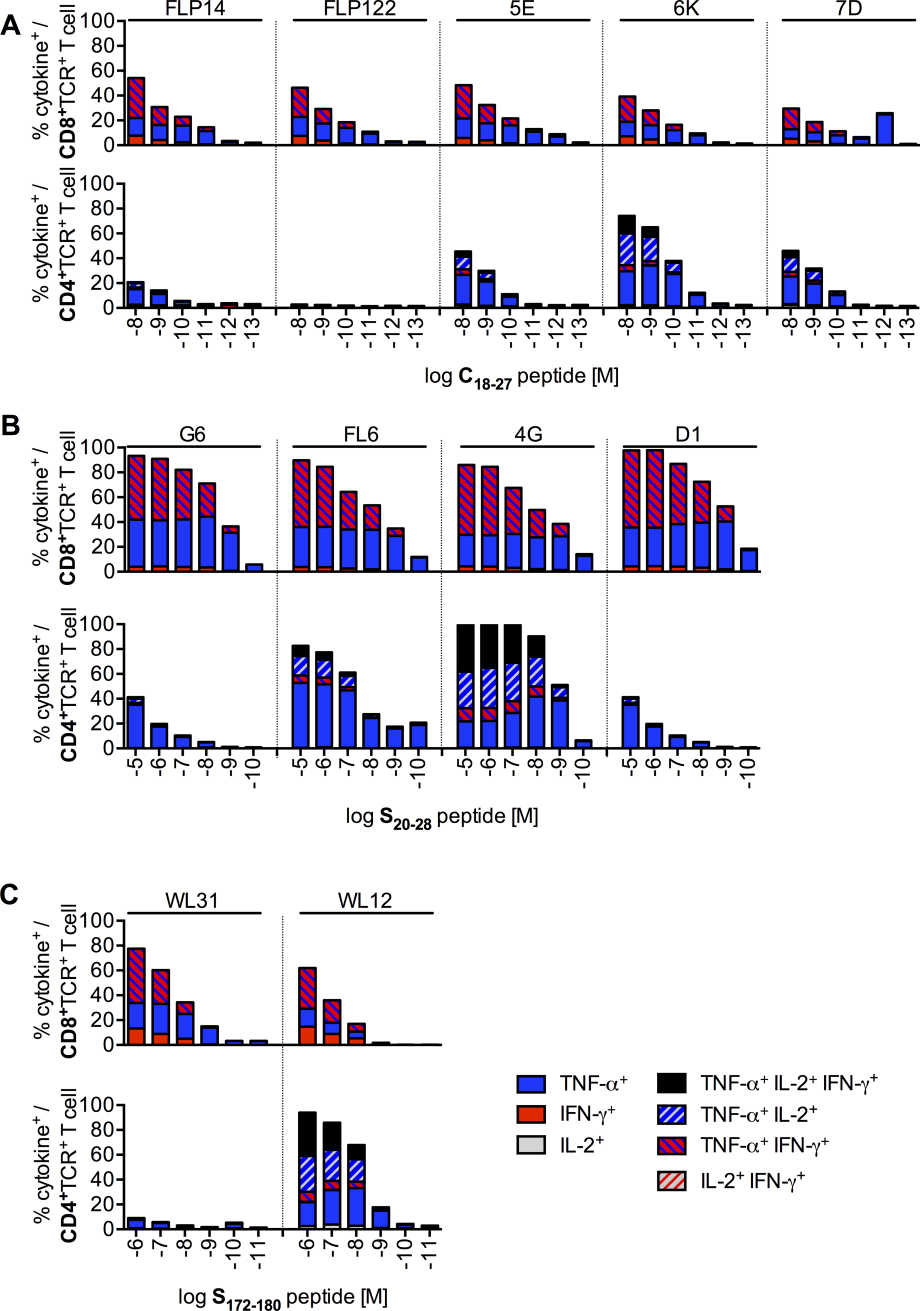
Polyfunctionality of TCR-transduced T cells. After retroviral transduction, CD8^+^ and CD4^+^ T cells were separated by magnetic activated cell sorting (MACS). Transduced CD8^+^ T cells were identified with MHC Streptamers and CD4^+^ T cells with an antibody against the murine constant domain of the transduced TCR. 1x10^5^ T2 cells loaded with decreasing amounts of peptide were co-cultured with 1x10^5^ CD8^+^ or CD4^+^ T cells expressing (A) C18-specific, (B) S20-specific, or (C) S172-specific TCRs. Cytokine^+^ cells were detected by intracellular cytokine staining and are given as % of TCR-transduced T cells. Data are presented as mean values from triplicate (CD8^+^) or single (CD4^+^) co-cultures.

To study the functional profile and sensitivity that our TCRs conferred to transduced T cells, we loaded T2 cells with decreasing amounts of peptide and co-cultured them with TCR^+^ T cells. All TCRs conferred polyfunctionality in terms of cytokine secretion to transduced CD8^+^ T cells and–except FLP122 and WL31 –also to CD4^+^ T cells (Fig 2). In both T-cell subsets tumor necrosis factor (TNF)-α was the predominantly produced cytokine. In addition to TNF-α, CD8^+^ T cells produced IFN-γ but not interleukin (IL)-2. CD4^+^ T cells expressed TNF-α, IFN-γ and IL-2 (Fig 2). Except for TCRs 6K (Fig 2A), 4G (Fig 2B) and WL12 (Fig 2C), TCR-transduced CD8^+^ T cells were more sensitive than CD4^+^ T cells to the presentation of HBV peptides on HLA-A*02. C18-specific T cells showed a minor cross-reactivity towards the S20 peptide (S4A Fig), whereas S-specific T cells were only activated by their cognate peptide (S4B and S4C Fig). Notably, staining of TCRs on CD4^+^ T cells with MHC multimers did not predict functionality: TCR FLP122 could be stained by the HLA-A*02_C18_ multimer, but was not functional (Fig 1D and Fig 2A). In contrast, FL6 did not bind the HLA-A*02_S20_ multimer, but delivered a strong activation signal to the T cell (Fig 1D and Fig 2B).

### Transduction with HBV-specific TCRs renders CD4^+^ and CD8^+^ T cells cytotoxic

**Fig 3:**
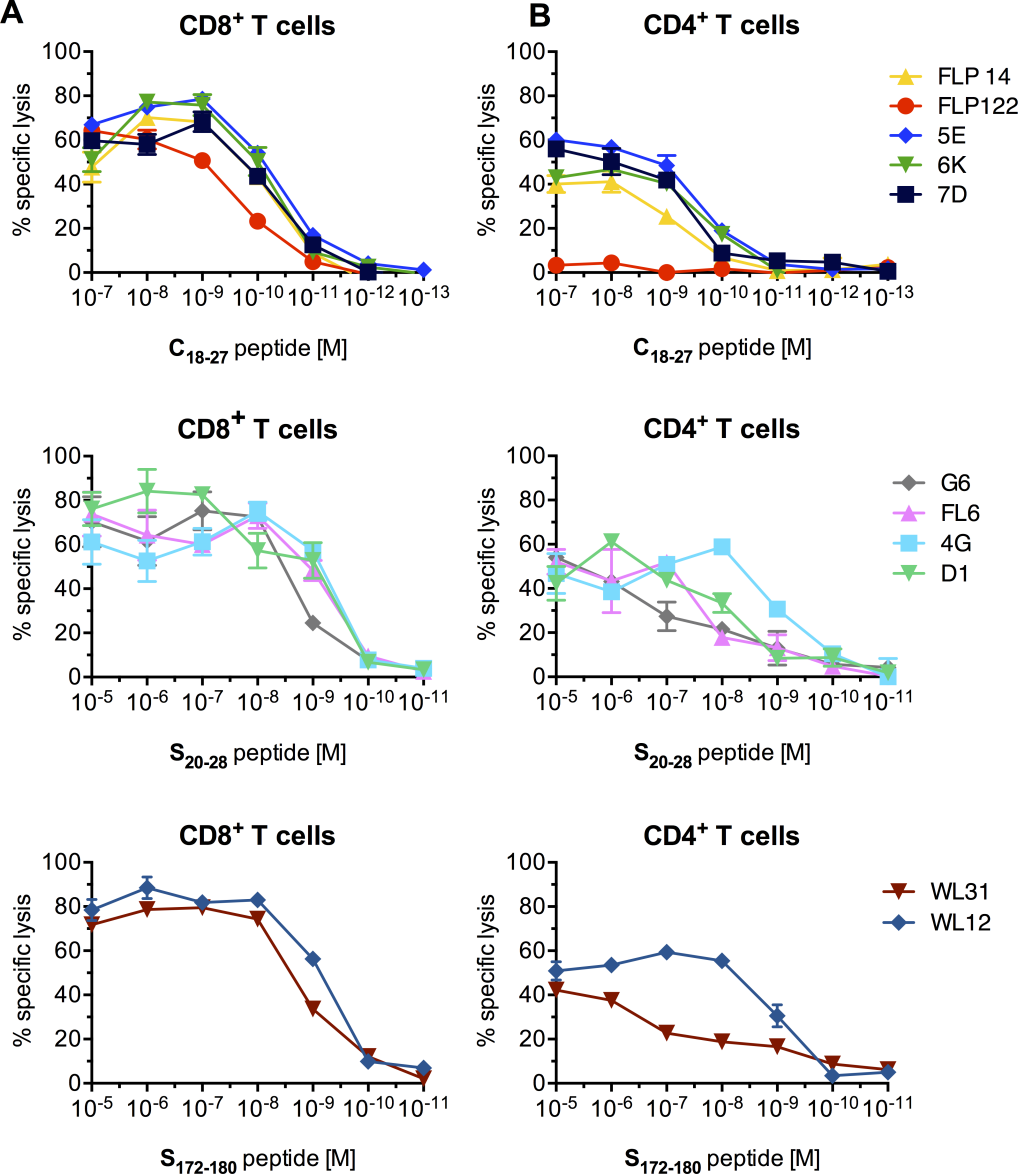
Sensitivity of TCR-transduced T cells. TCR-transduced CD8^+^ (A) or CD4^+^ (B) T cells expressing C18-specific, S20-specific, or S172-specific TCRs were co-cultured with T2 cells pulsed with decreasing peptide concentrations (effector to target ratio, E:T = 1:1). Each color represents one TCR. Cytotoxicity of effector cells was assessed by chromium release assay. Data are presented as mean values +/- SEM from triplicate co-cultures.

To further dissect TCR functionality, we analyzed the killing capacity of T cells transduced with our HBV-specific TCRs (Fig 3). Hereby, C18-specific TCRs conferred the highest sensitivity. With a half maximum lysis at a peptide concentration of below 100 pM, C18-specific cells were about ten-fold more sensitive than S20- or S172-specific CD8^+^ T cells (Fig 3A). The killing profile of CD8^+^ T cells was similar when they belonged to a group with the same peptide specifictiy (Fig 3A). Total lysis by CD4^+^ T cells was reduced by approximately 20% compared to CD8^+^ T cells expressing the same TCR (Fig 3B). Nevertheless, most of the TCRs converted not only CD8^+^ but also CD4^+^ T cells to MHC-I-restricted T effector cells and mediated lysis of target cells (Fig 3B). Exceptions were the TCRs FLP122 and WL31 transduced into CD4^+^ T cells, which showed only poor lysis of target cells (Fig 3B, S3 Table) in accordance with the low cytokine production described above (Fig 2A and 2C).

Overall, nine of the 11 TCRs had a high functional avidity and their expression resulted in a polyfunctional response of the transduced T cells upon antigen recognition, comprising cytokine production and cytotoxicity in CD8^+^ as well as CD4^+^ T cells.

### HBV-specific TCRs recognize peptides from different HBV genotypes presented on various HLA-A*02 subtypes

With regard to a potential clinical application of HBV-specific TCRs for T-cell therapy it is of interest to have TCRs available for a broad spectrum of patients. The HLA background of all our original donors was HLA-A*02:01 (Table 1) and they were stimulated with peptides derived from HBV genotype (gt) D. Therefore, we first addressed the suitability of our TCRs to recognize peptides presented on other HLA-A*02 subtypes using lymphoblastoid cell lines (LCL).

**Fig 4:**
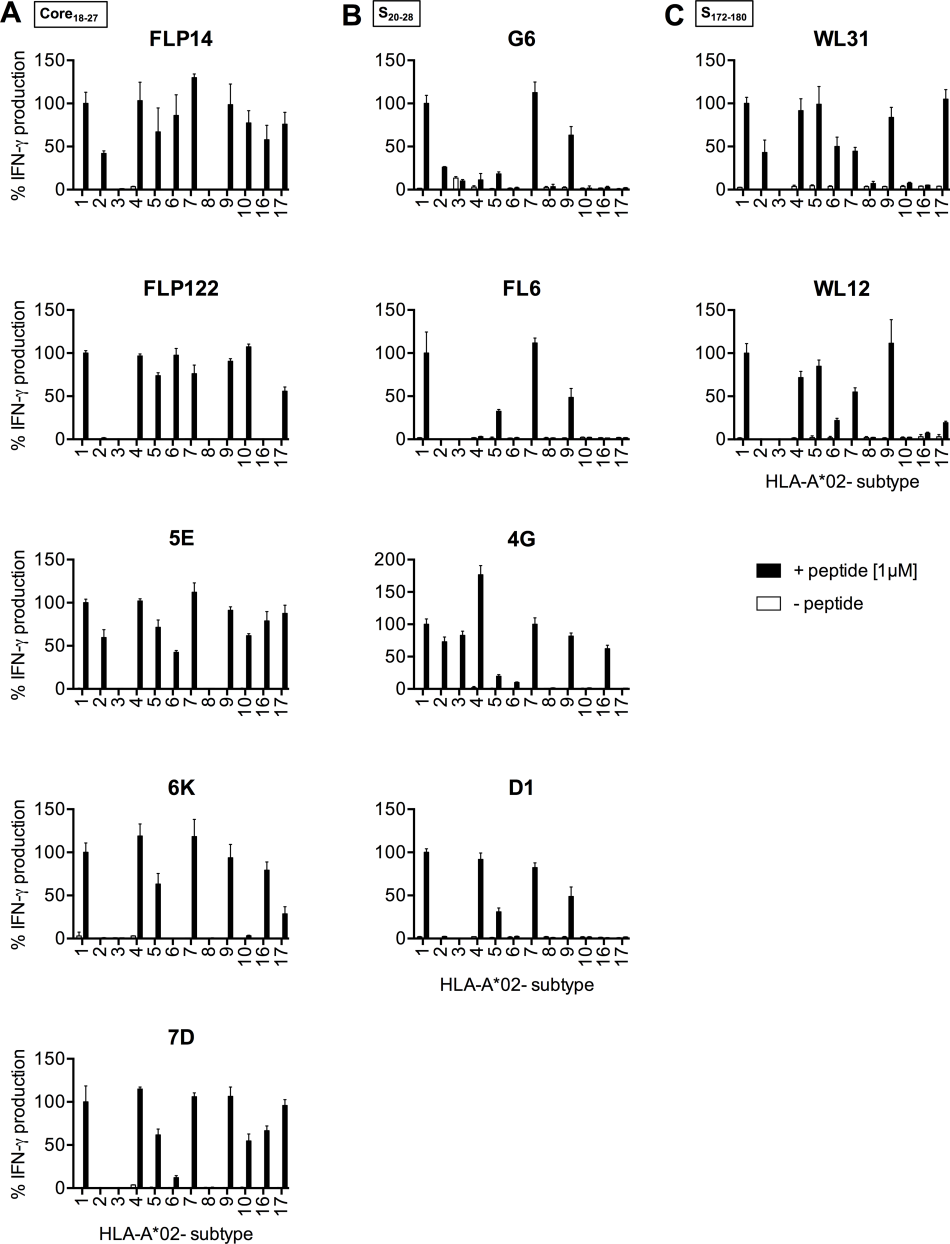
Recognition of different HLA-A*02 subtypes. LCL with varying HLA-A*02 subtypes were loaded with HBV peptides (1 μM) and co-cultured with TCR-transduced C18-specific (A), S20-specific (B) or S172-specific (C) CD8^+^ T cells (E:T 1:1). T-cell activation was measured by IFN-γ ELISA. All donors, from which the TCRs originated, carried the HLA-A subtype *02:01 and therefore data were normalized to the recognition of the LCL with subtype *02:01 (set to 100%). The x-axis indicates the HLA-A*02 subtype of the target cells used (HLA-A*02:xx). Data are presented as mean values +/- SEM from triplicate co-cultures.

C18 peptide was recognized by the C18-specific TCRs FLP14 and 5E when presented on 10/12 HLA-A*02 subtypes tested, on 8/12 by FLP122 and 7D, and on 7/12 by 6K (Fig 4A). The S20-specific TCR 4G recognized presentation of peptide on 9/12, TCRs G6 and D1 on 5/12, and FL6 on 4/12 HLA-A*02 subtypes, respectively (Fig 4B). The S172-specific TCR WL31 recognized the peptide on 8/12 and WL12 on 7/12 HLA-A*02 subtypes (Fig 4C). The LCL expressing HLA-A*02:03 was only able to activate S20-specific TCR 4G (Fig 4B). Of note, a stronger T-cell activation via a TCR in the previous experiments did not correlate with the number of different HLA-A*02 subtypes recognized.

**Fig 5:**
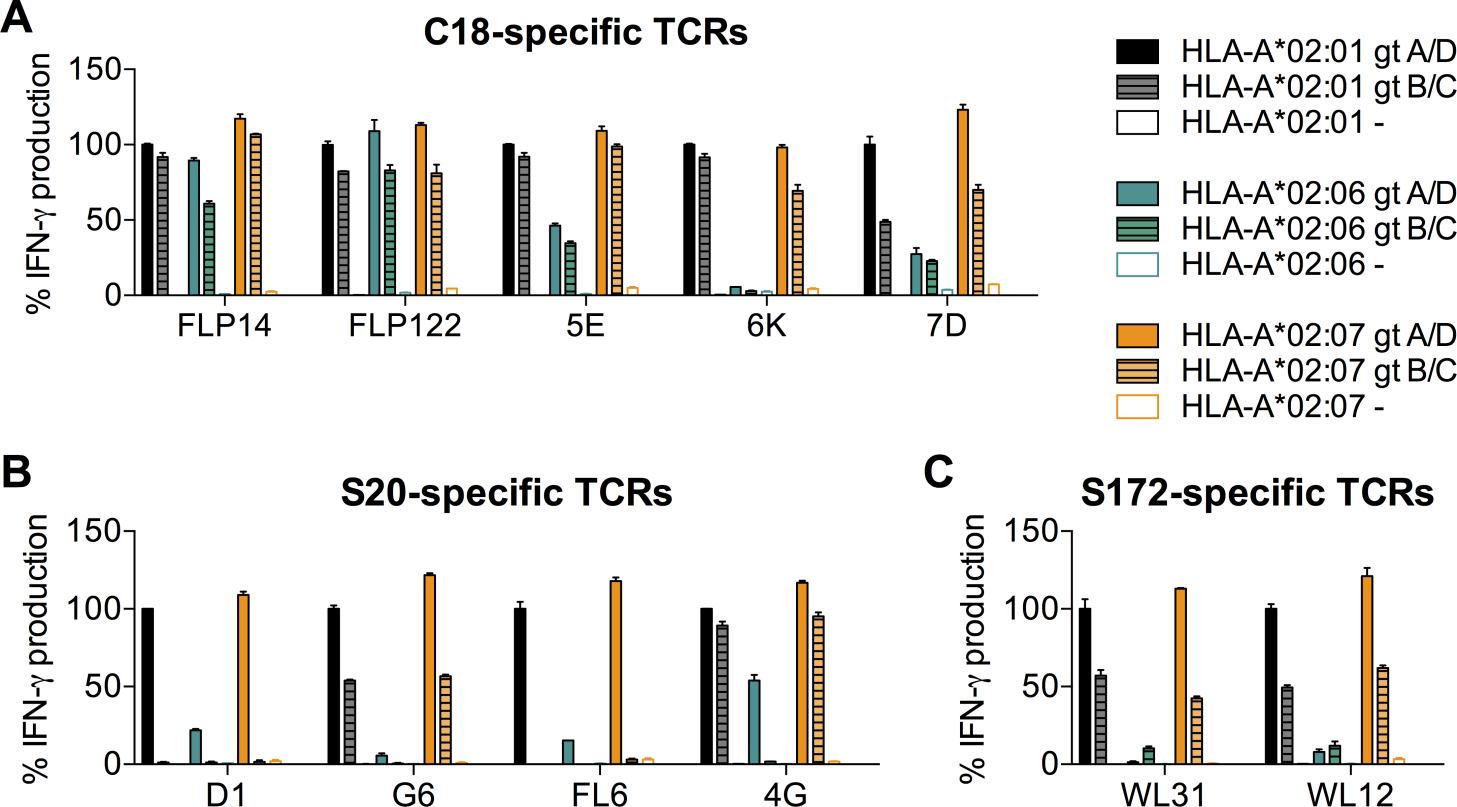
Recognition of different HBV genotypes. LCL with varying HLA-A*02 subtypes were loaded with HBV peptides (1 μM) and co-cultured with T cells (CD8^+^ and CD4^+^ combined) transduced with C18-specific (A), S20-specific (B) or S172-specific (C) TCRs. T-cell activation was determined by IFN-γ secretion measured by ELISA. IFN-γ secretion is shown relative to that determined upon recognition of subtype HLA-A*02:01 loaded with the respective genotype A/D peptide (set to 100%), because this constellation had been used for isolating the TCRs. Peptide sequences: C18: HBV gt A/D: FLPSDFFPSV, HBV gt B/C: FLPSDFFPS**I**; S20: HBV gt A/D: FLLTRILTI, HBV gt B/C: FLLT**K**ILTI; S172: HBV gt A/D: WLSLLVPFV, HBV gt B/C: WLSLLV**Q**FV. Data are presented as mean values +/- SEM from duplicate co-cultures.

In addition, we analyzed whether our TCRs would recognize the corresponding peptides derived from HBV gt B and C, which are dominant in Asia, on the most frequent HLA-A*02 subtypes *02:01, *02:06 and *02:07. All C18- and all S172-specific TCRs, as well as the S20-specific TCRs G6 and 4G recognized their cognate peptides derived from HBV gt A, B, C or D not only when presented on HLA-A*02:01, but also when presented on *02:07 (Fig 5A–5C). On HLA-A*02:06, all C18-TCRs recognized the gt A- and D-variant of the C18 peptide, and all but 6K the B- and C-variant (Fig 5A). In contrast, S20 and S172 peptides were only weakly recognized by any of our TCRs on HLA-A*02:06 except 4G, which recognized at least the gt A- and D-variant of S20. Thus, HBV gt A- and D-derived peptides were recognized on all HLA-A*02 subtypes except *02:08, and HBV gt B and C peptides on HLA-A*02:01, *02:06, and *02:07 by at least one of our TCRs.

### TCR-transduced T cells recognize endogenously processed peptide

**Fig 6:**
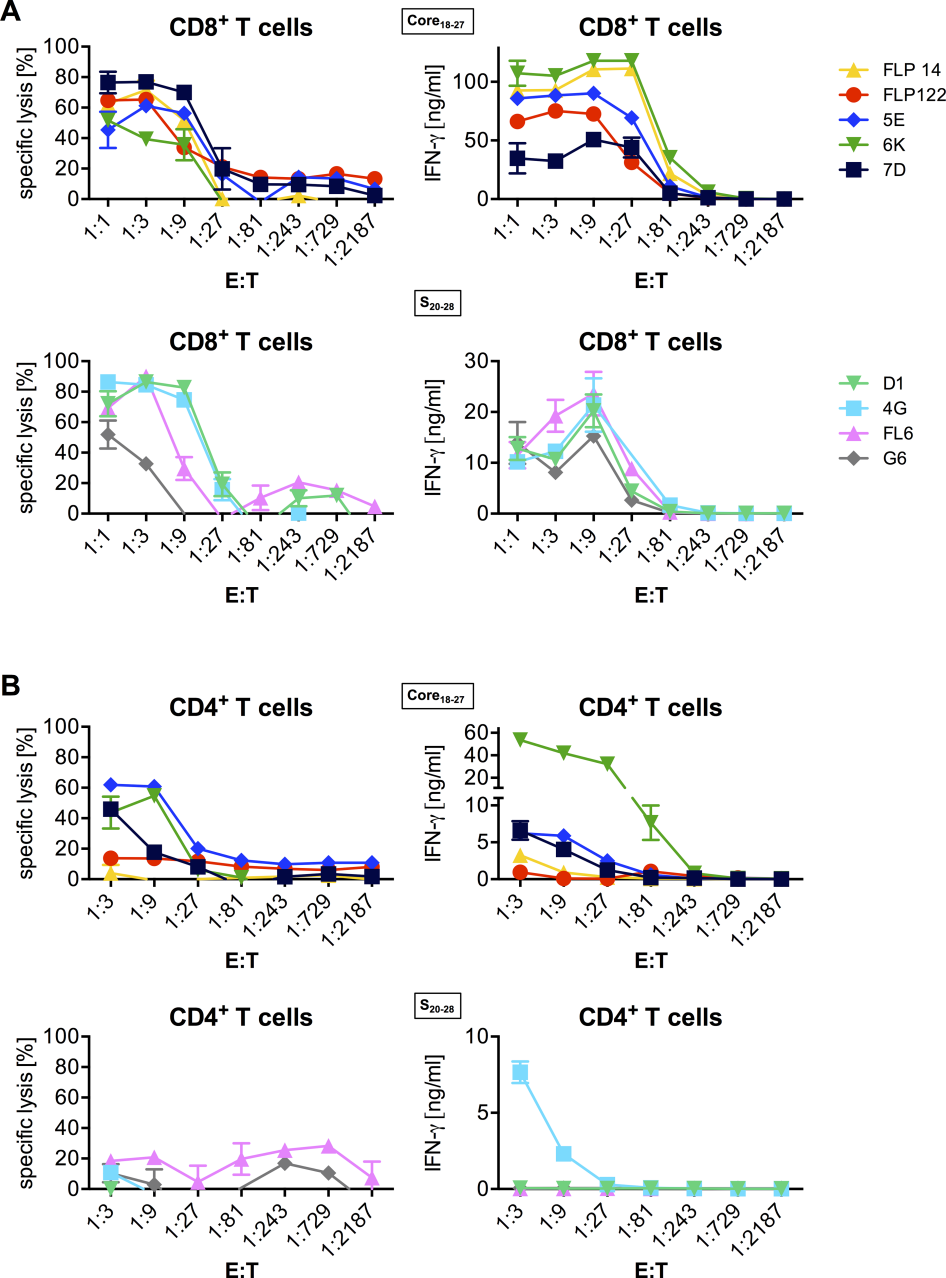
Recognition of endogenously processed HBV peptides by TCR-transduced T cells. After retroviral transduction with respective TCRs, CD8^+^ and CD4^+^ T cells were separated by MACS. Specific lysis of HBV-replicating HepG2.2.15 hepatoma cells or T-cell activation (indicated by IFN-γ secretion) by TCR-transduced CD8^+^ (A) or CD4^+^ (B) T cells was measured. The x-axis indicates the decreasing ratio of TCR^+^ effector to target cells. Each color represents one TCR. Data are presented as mean values +/- SEM from triplicate co-cultures.

External loading of peptides on target cells allows quantifying the magnitude of a T-cell response to a defined peptide concentration. However, *in vivo* HBV proteins will be processed within the infected cell and will be loaded on MHC-I molecules in the endoplasmatic reticulum. To investigate whether endogenously processed HBV peptides are recognized by our TCRs, we co-cultured TCR-transduced T cells with an HLA-A*02^+^ human hepatoma cell line that replicates HBV. CD8^+^ T cells expressing any C18- or S20-specific TCR killed HBV-replicating HepG2 cells and secreted high amounts of IFN-γ, even at relatively low effector to target (E:T) cell ratios (Fig 6A). They did not react unspecifically to the parental cell line that does not contain HBV (S5 Fig). Expression of the C18-specific TCR 5E, 6K or 7D (all derived from donor 1, who had resolved his HBV infection), but not of TCR FLP14 or FLP122 (derived from donor 2, who had a protracted course of HBV infection), conferred effector function also to CD4^+^ T cells enabling them to specifically kill HBV-replicating HepG2 cells (Fig 6B, S5 Fig). In contrast, S20-specific TCRs (except TCR 4G) were not able to activate CD4^+^ T cells (Fig 6B). S172-specific T cells were not activated at all upon co-culture with HBV-positive HepG2 hepatoma cells, but were activated by epithelial cells that had been transfected with an S-plasmid (S6 Fig). This indicates a certain defect in antigen processing or presentation of this peptide in HepG2 hepatoma cells, but still allows to conclude that processed antigen may be recognized [23]. Thus, all HBV-specific TCRs were able to detect HBV peptides presented after intracellular processing.

### Treatment of HBV-infected cells with gene-modified T cells leads to a rapid reduction of viral markers

**Fig 7:**
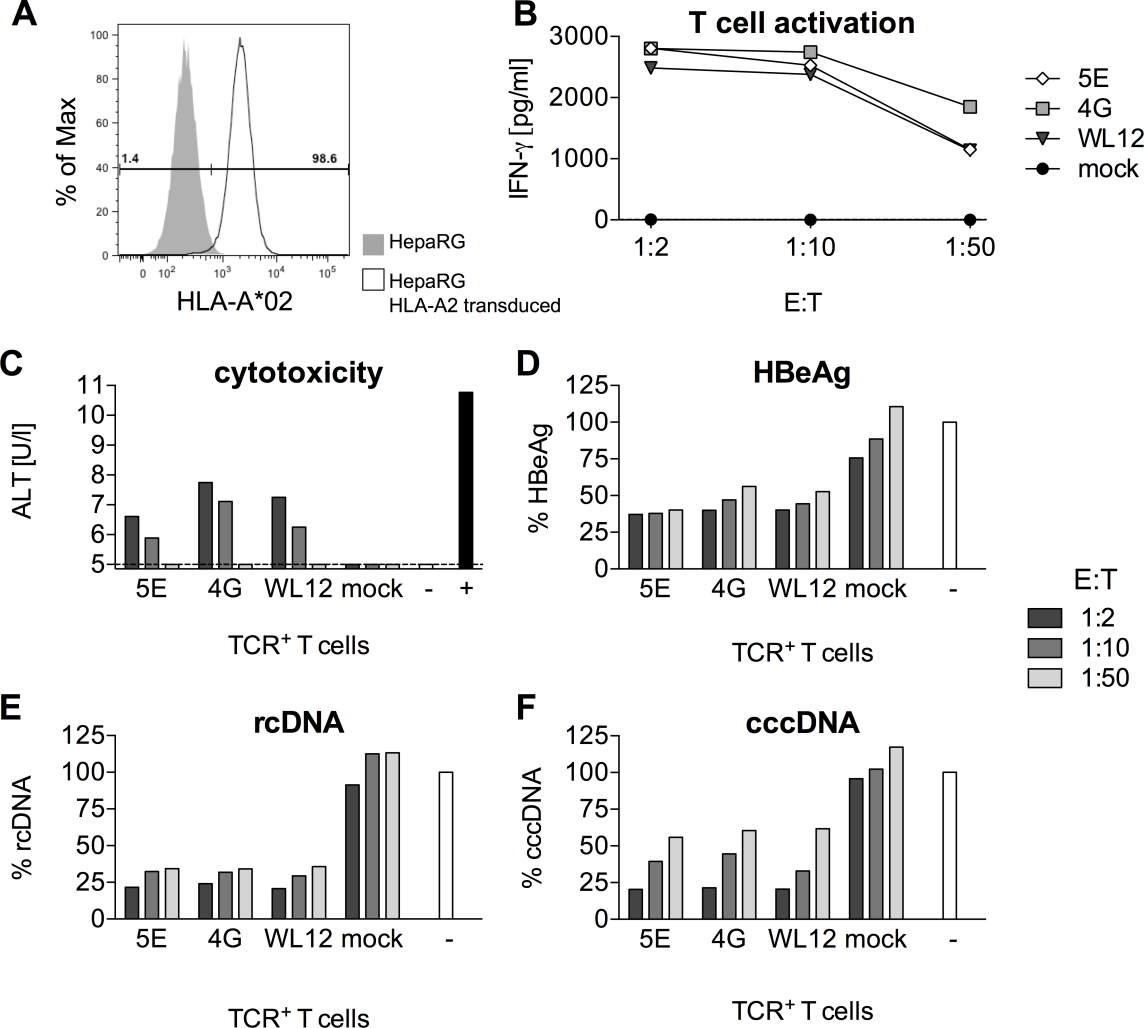
Recognition of HBV-infected cells by TCR-transduced T cells. (A) Expression of HLA-A*02 on HepaRG target cells determined by flow cytometry. (B-D) HBV-infected HepaRG cells were co-cultured with decreasing numbers of TCR-transduced T cells for 44 hours. One TCR per peptide specificity was selected (5E: C18-specific, 4G: S20-specific, WL12: S172-specific). Mock = untransduced PBMC,— = medium control without PBMC. (B) T-cell activation measured by release of IFN-γ into the supernatant. (C) Alanine transaminase (ALT) released from dying hepatocytes. Supernatants from triplicate co-cultures per TCR were pooled for this analysis. + = Triton-X, positive control for cell lysis. (D) HBeAg in the supernatant was measured using a diagnostic ELISA. (E) Intracellular viral rcDNA and (F) nuclear cccDNA on day two were determined using qPCR. Values were normalized to the untreated medium control. Data are presented as mean values +/- SEM from triplicates.

Our next step was to assess the antiviral capacity of TCR-transduced T cells (CD8^+^ and CD4^+^ combined). To this end, we generated a stable HLA-A*02 expressing HepaRG cell line (Fig 7A), which allows infection with HBV. In this set-up, only one TCR for each peptide specificity was tested. TCRs 5E (C18-specific), 4G (S20-specific) and WL12 (S172-specific) were selected according to the combined results of functional avidity and recognition of HLA-A*02 subtypes in our previous experiments (S3 Table). TCR-transduced T cells were specifically activated by HBV-infected cells to produce IFN-γ (Fig 7B). Specific lysis of infected cells was indicated by the release of the hepatocyte enzyme alanine transaminase (Fig 7C). Most importantly, TCR-transduced, re-directed T cells were able to rapidly reduce secreted (HBeAg) and intracellular HBV rcDNA (Fig 7D and 7E). Reduction of cccDNA, which is the episomal persistence form of HBV in the nucleus of infected cells, required target cell killing and was directly dependent on the T cell to target cell ratio (Fig 7F). Total DNA, in contrast, was already reduced at an E:T ratio as low as 1:50, indicating additional non-cytolytic control by T-cell cytokines. Taken together, all T cells genetically engineered to become HBV-specific were able to reduce HBV replication and kill infected cells independently of their peptide specificity.

## Discussion

In this report, we isolated 11 TCRs that are specific for three different HBV peptides presented on HLA-A*02 (Core_18-27_, S_20-28_, S_172-180_). Using genetic engineering, the HBV-specific TCRs could be expressed on the surface of CD8^+^ and CD4^+^ T cells and induced typical T-cell effector functions upon specific recognition of exogenously added peptides and endogenously processed antigens.

HBV-specific T cells can be detected by MHC multimer staining at frequencies of about 0.1–1.1% in acute HBV infection, but not in chronic hepatitis B patients [24]. We therefore isolated TCRs from donors with either acute or resolved HBV infection. The TCRs with the highest functional avidity originated from memory T cells that had probably undergone a selection for high avidity TCRs due to repeated exposure to antigen [25] in the donor who had resolved the infection. TCRs isolated from donors with acute, resolving hepatitis B by contrast had a lower functional avidity.

Among the identified TCR chains we could detect a common usage of Vβ12 for recognition of the S20 peptide presented on HLA-A*02:01. The fact that the Vβ12 chain of two TCRs from different donors had almost identical CDR3 regions could indicate the existence of a public TCR as described for other viral infections [26]. Although a skewed TCR Vβ gene usage was found in patients who had recovered from acute hepatitis B [27], Vβ12 usage was not reported. This could be attributed to the cohort containing only few subjects with HLA-A*02:01 [27], which might also influence the choice of TCR variables because their CDR1 and CDR2 regions interact with the MHC molecule [28]. Hence, whether Vβ12 usage is associated with recovery from hepatitis B in HLA-A*02:01 subjects remains to be confirmed in larger cohorts.

One challenge of TCR gene therapy is the potential mispairing of endogenous and introduced TCR chains, which could result in autoimmunity. We therefore improved TCR expression and specific pairing by murinization of constant domains, codon-optimization, inserting additional disulfide bonds, and ensuring equimolar translation of both TCR chains from one construct. All of these measures have been shown to greatly reduce the risk of mispairing of endogenous and introduced TCR chains [29–31]. As a consequence, our transduced T cells did not react to HBV-negative cells, which only present peptides from self-antigens.

In general, cross-reactivity of the transduced, correctly paired TCRs is highly unlikely as they were isolated from patients controlling HBV and underwent thymic selection. Recognition of self-antigens by the TCRs would hence have caused autoimmunity in the donors, which remained healthy after resolution of HBV. In our experiments, however, we detected a cross-reactivity of C18-specific T cells against high peptide concentrations of the S20 peptide but not against other control peptides tested. We assume that this is an exclusive phenomenon that might even increase sensitivity of “C18-specific” T cells towards HBV-infected cells.

The strength of a T-cell response correlates with TCR avidity [14]. The functional avidity of our TCRs was in the picomolar range, which is similar to or even higher than what has been reported for high-avidity TCRs against other viral antigens [21,32]. Sensitivity of TCRs directed against the core epitope C18 was higher than that against S-derived peptides. While S-specific responses are also associated with resolution of infection [33], C18-specific CD8^+^ T cells represent the dominant effector cell population in patients with acute, resolving hepatitis B [34,35]. This may be due to a high binding affinity of C18 to HLA-A*02:01 (IC_50_ 2.5 nM) [36] leading to a better presentation and hence to a more effective T-cell response [37].

Polyfunctionality of T cells is associated with long-term control of viral infections [38]. Our data demonstrate that T cells redirected to express HBV-specific receptors are polyfunctional in terms of secretion of multiple cytokines and cytotoxicity. Treatment of HBV-infected HepaRG cells with TCR-transduced T cells lead to a rapid reduction of the HBV persistence form, the viral cccDNA, which can be explained both by killing of infected cells or by its cytokine-induced non-cytolytic removal after APOBEC3-induced deamination [39,40]. However, after two days, the co-culture with T cells had to be ended because HepaRG cells did not tolerate the change of medium. Hence, a more robust target cell line that can be infected with HBV is necessary to analyze the antiviral activity of TCR-transduced T cells [41]. In this setting, TCR-transduced T cells could be used as an experimental tool not only to define their antiviral mechanisms, but also to study virus-host cell interactions that may for example result in alteration in immune pathways and antigen presentation.

Efficacy of adoptive T-cell therapy is likely to be enhanced, if antigen-specific CD4^+^ T cells are co-infused to provide help to cytotoxic CD8^+^ T cells [17]. A prerequisite for MHC-I-restricted TCRs to function in CD4^+^ T cells is that the TCR is of high affinity and does not need CD8 co-receptor binding [18]. We found several TCRs specific for HBV antigens that were functional without CD8 co-receptor expression arguing for a strong interaction of MHC and TCR. However, MHC multimer staining did not predict functionality of TCR-transduced CD4^+^ T cells. A discrepancy between MHC multimer binding and effector function [42] but not TCR-ligand K_off_-rate [43] has been reported for CD8^+^ T cells and might be explained by the density of the MHC:peptide complex [44] or additional molecules involved in formation of the immunological synapse.

Binding of the TCR to its cognate peptide depends on effective presentation by the MHC-I molecule. We found that TCRs, which were isolated from HLA-A*02:01 donors, were able to recognize peptides presented on other HLA-A*02 subtypes. This is of interest for a clinical application of T-cell therapy, since a broader range of HLA-A*02 subtypes recognized will increase its applicability. Importantly, TCRs should recognize the HLA alleles most common in populations where hepatitis B is endemic. For instance, up to one third of the Chinese population carry the alleles HLA-A*02:03, *02:06, or *02:07 (www.allelefrequencies.net). Our C18-specific TCRs could recognize *02:06 and *02:07, but not *02:03 bound C18 peptide. Although a high binding affinity of C18 to *02:03 was predicted in a molecular binding assay [36], analysis of the crystal structure revealed a steric hindrance between the *02:03 binding groove and the C18 peptide resulting in reduced thermostability [45]. Remarkably enough, while a T-cell response to S20 was only found for *02:01 in a Chinese cohort [33], our high-avidity TCR 4G recognized S20 also when presented on HLA-A subtype *02:03 or *02:07. Importantly, most of our TCRs recognized peptides from HBV gt A and D strains, most frequent in America and Europe, but also peptides from HBV gt B and C, most frequently found in Asia [33], indicating that the isolated TCRs could also be applied to a significant number of Asian patients.

As an alternative approach, HBV-specific CARs could be used, which would be applicable irrespective of the patient’s MHC haplotype [8]. While their broader applicability and a non-existent risk for mispairing with endogenous TCR chains are clear advantages, their antibody-based antigen-recognition domain is usually not as sensitive as a natural T-cell receptor and needs higher antigen loads for T-cell activation.

In conclusion, we identified TCRs with a high functional avidity for HBV S- and core-derived peptides with a comprehensive analysis of a unique set of TCRs. They can be employed as an experimental tool to study immune pathways in viral diseases ranging from antigen recognition to antiviral mechanisms exerted by TCR-transduced T cells. Since the TCRs are functional in CD8^+^ as well as CD4^+^ T cells and recognize peptides presented on a broad range of HLA-A*02 molecules, they are interesting for clinical application of adoptive T-cell therapy.

## Materials and methods

### T-cell stimulation

PBMC from HLA-A*02^+^ donors with an acute or resolved HBV infection were isolated via a standard Ficoll gradient. Informed consent in writing was obtained from each patient. T2 cells were pulsed with 1 nM or 1 μM of peptide (C18-27: FLPSDFFPSV, S20-28: FLLTRILTI, S172-180: WLSLLVPFV, JPT Peptide Technologies, Berlin, Germany) for 2 hours at 37°C. Then, they were irradiated with 35 Gy, washed 2 times and adjusted to the desired cell concentration. Complete PBMC or CD8^+^ T cells, purified with Dynabeads Untouched (Life Technologies, Darmstadt, Germany), were incubated with peptide-loaded T2 cells for 7–14 days and restimulated for another 7 days according to S1 Table. 10 ng/ml IL-7 and IL-15 (Peprotech, Hamburg, Germany) was added on day 0, and 50 U/ml Proleukin (Novartis Pharmaceuticals, West Sussex, UK) were added on days 1, 4, 8 and 11. PBMC were kept in T-cell medium (TCM): RPMI, 10% human serum (own production from male, healthy donors), 1% pen/strep, 1% glutamine, 1% sodium pyruvate, 1% non essential amino acids, 10 mM HEPES, 16,6 μg/ml Gentamycin (all from Life Technologies).

### T-cell cloning

T cells were stained with HLA-A*02/Core_18-27_, HLA-A*02/S_20-28_ or HLA-A*02/S_172-180_ Streptamer (Institute of Microbiology, Immunology and Hygiene, Munich, Germany) corresponding to the peptide used for stimulation. 1 μg of MHC-I and 0.75 μg of Strep-Tactin PE (IBA, Göttingen, Germany) were incubated with the cells in 50 μl of FACS buffer for a total of 45 min. After 20 min, antibodies for surface staining were added. MHC Streptamer^+^ CD8^+^ T cells were enriched using a MoFlo legacy cell sorter (Beckmann Coulter). Cells were sorted into 10 μM Biotin/FCS to remove the MHC Streptamer. 0.3–0.5 cells/well were seeded in 96-well round bottom plates containing 7.5x10^4^ irradiated heterologous PBMC (35 Gy), 1x10^4^ LCLs (50 Gy), 50 U/ml IL-2 and 30 ng/ml OKT-3 antibody (eBioscience, Frankfurt, Germany). For expansion T-cell clones were moved to a 12-well plate containing 5x10^6^ irradiated PBMC, 1x10^6^ LCLs and 30 ng/ml OKT-3. 50 U/ml IL-2 were supplemented on day 1, 5, 8 and 11.

### Analysis of TCR repertoire

For RNA extraction from T-cell clones, Trizol (Life Technologies) was used according to the manufacturer’s instructions including 1-bromo-3-chloropropane (Sigma-Aldrich, Taufkirchen, Germany) and 20 μl Linear Acrylamide (Life Technologies). RNA was reverse transcribed to cccDNA using Superscript II (Life Technologies) or AMV RT (Roche, Mannheim, Germany) with the CA1 primer specific to the TRAC (5’-AGACCTCATGTCTAGCACAG-3’). TCR chains were amplified from cDNA with Illustra PureTaq PCR Beads (GE, Freiburg, Germany) using degenerated primers, VPANHUM (5’-TGAGTGTCCCPGAPGG2P-3’) and CA2 (5’-GTGACACATTTGTTTGAGAATC-3’) for α chains, VP1 (5’-GCIITKTIYTGGTAYMGACA-3’) or VP2 (5’-CTITKTWTTGGTAYCIKCAG-3’) and CP1 (5’-GCACCTCCTTCCCATTCAC-3’) for β chains. Blasting the sequencing results with IMGT/V-QUEST identified TCR chains.

### Cloning of TCR chains

5’ Primers including a Kozak sequence and a NotI restriction site were designed according to the variable region identified for each TCR. An EcoRI or BsrGI restriction site was added to the 3’ primers for the constant regions: TRAC (TRAC-EcoRI 5’-GGAATTCTCAGCTGGACCACAGCCGCAGC-3’ and TRAC1-BsrGI 5’-CTTGTACATCAGCTGGACCACAGCCGCAGC-3’) or TRBC (TRBC1-EcoRI 5’-TGGAATTCTCAGAAATCCTTTCTCTTGACC-3’ and TRBC2-EcoRI 5’-TGGAATTCCTAGCCTCTGGAATCCTTTCTC-3’). TCR chains were amplified from cDNA with Phusion Hot Start II (NEB, Frankfurt, Germany) and cloned separately into the retroviral vector MP71 [46]. TCR chains were also codon-optimized and synthesized at GeneArt (Regensburg, Germany), fused by a P2A element and substituted with murine constant domains as described previously [47] (S3 Fig).

### Flow cytometry

Staining was done for 30’ on ice in the dark using primary antibodies (eBioscience or BD Biosciences, Heidelberg, Germany) diluted in FACS buffer (0.1% BSA/PBS). Transduction efficiency was assessed one day after the 2nd transduction by staining the TCR with MHC Streptamers (see above) or an anti-mTRBC antibody. For intracellular cytokine staining, Brefeldin A (8 μg/ml, Sigma-Aldrich) was added for 4 hours during antigen stimulation. After staining of dead cells with EMA (Life Technologies) and cell surface molecules, intracellular cytokines were stained using the Cytofix/Cytoperm Kit (BD Biosciences). CD4^+^ T cells were stained with antibodies against IL-2, IFN-γ and TNF-α; CD8^+^ T cell with antibodies against IFN-γ and TNF-α, but not IL-2 as it was hardly detected in our previous experiments. Cells were analyzed using a FACSCanto II flow cytometer (BD Biosciences) and data were analyzed with FlowJo 9.2 software.

### Retroviral transduction

Plasmids were amplified using Stbl3 bacteria (Life Technologies) and purified with a Midiprep Plasmid DNA Endotoxin-free Kit (Sigma-Aldrich). 293T cells were transfected in a 6 well plate with 4 μg of plasmid DNA (2 μg TCR plasmids, 1 μg pcDNA3.1-MLVg/p, 1 μg pALF-10A1-MLVenv) and 10 μl of Lipofectamin 2000 (Life Technologies). After 6 hours, the medium was replaced with full DMEM medium. After 24 and 48 hours, the retrovirus supernatant was collected and filtered through a 0.45 μm filter. PBMC were pre-stimulated for 2 days in TCM supplemented with 300 U/ml IL-2 on non-tissue culture plates, which were coated with 5 μg/ml OKT-3 and 0.05 μg/ml anti-CD28 antibody (eBioscience) for 2 hours at 37°C, blocked with 2% BSA for 30 min and washed with PBS. Retrovirus supernatant was centrifuged at 2000 x g, 32°C for 2 hours on non-tissue culture plates coated with 20 μg/ml RetroNectin (Takara, St. Germain en Laye, France). The retrovirus supernatant was removed and PBMC were spinoculated on the virus-coated plate at 1000 x g for 10 min. A second transduction was performed after 24 hours.

### Co-culture with T2 cells

CD8^+^ and CD4^+^ T cells were isolated out of total transduced PBMC via positive selection magnetic activated cell sorting (MACS, Miltenyi, Bergisch-Gladbach, Germany). For intracellular cytokine staining 1x10^5^ TCR^+^ T cells were incubated with 1x10^5^ peptide-loaded T2 cells. After 1 hour, Brefeldin A was added to arrest cytokine secretion.

### Chromium release assay

T2 cells were incubated with the indicated concentration of peptide and 50 μCi of 51^Cr^ for 1 hour at 37°C and washed 2.5 times. 2x10^4^ target cells were co-cultured with 2x10^4^ T-cell clones or TCR-transduced T cells (E:T ratio = 1:1). The spontaneous (SL) and maximum lyses (ML) were assessed with medium or 2% Triton-X instead of effector cells. The cells were incubated for 4 hours at 37°C, spun down, and the supernatants were transferred to LUMA-plates (Perkin Elmer, Rodgau, Germany). Radioactivity released into the supernatant was measured with Top Count NXT (Perkin Elmer). Specific lysis was calculated: (counts sample–counts SL)/(counts ML–counts SL)x100.

### Co-culture with LCL

Lymphoblastoid cell lines carrying a subtype of HLA-A*02 were used: JY A*02:01, Bello A*02:02, CF160 A*02:03, RML A*02:04, WT49 A*02:05, CLA A*02:06, TAB089 A*02:07, KLO A*02:08, OZB A*02:09, XLI-ND A*02:10, TUBO A*02:16, AMALA A*02:17. LCL were loaded with 1 μM or decreasing amounts of peptide for 2 hours at 37°C and then washed 2.5 times. 1x10^4^ TCR^+^ T cells were incubated with 1x10^4^ peptide-loaded LCL. After 72 hours, supernatants were taken and analyzed for IFN-γ by ELISA (BioLegend, London, UK).

### Co-culture with hepatoma cells

HepG2 and HepG2.2.15 hepatoma cells were kept in full DMEM medium (DMEM, 10% FCS, 1% pen/strep, 1% sodium pyruvate, 1% NEAA; Life Technologies). For co-culture experiments 5x10^4^ target cells per well were seeded in collagen-coated (Serva, Heidelberg, Germany) 96-well flat bottom plates. When cells had reached confluence, differentiation medium was applied (Williams medium, 5% FCS, 1% Pen/Strep, 1% sodium pyruvate, 1% NEAA and 0.5% DMSO (Sigma-Aldrich). Target cells were used for experiments after 10 to 14 days of differentiation. The number of effector T cells/well was adjusted according to the transduction efficiency of each receptor to identical numbers of TCR-expressing cells. Viability of target cells was assessed using an XTT assay (Roche) and supernatants were subjected to an IFN-γ ELISA (BioLegend).

### Generation of transgenic HepaRG cells

HLA-A2 was stably integrated into HepaRG cells (HLA-A*23:01, *29:02; HLA-B*44:03; HLA-C*04:24, *16:01; HLA-DRB1*07:01; HLA-DQB1*02:02; HLA-DPB1*04:01, 06:01) by retroviral transduction using the Clontech pQCXIN retroviral vector. In brief, the packaging cell line 293 T was transfected in a cell culture dish (9 cm diameter) with 10 **μ**g pQCXIN-HLA-A2 and 10 **μ**g of the packaging vector PCL-10A1 via calcium phosphate transfection. After 24 of incubation, the retrovirus supernatant was collected, filtered through a 0.45 **μ**m filter, mixed 1:1 with fresh HepaRG medium and supplemented with. HepaRG cells were transduced at the presence of 8 **μ**g/**μ**l polybrene. For selection of transduced cells 0.6 mg/ml, Geneticin was added one day after transduction. After 14 days, HLA-A2 expressing HepaRG cells were sorted at the FACS Aria and seeded in a limiting dilution to grow monoclonal cell lines. HLA-A2+ HeLa cells were generated accordingly.

### Co-culture with HepaRG cells

HepaRG cells were differentiated for 4 weeks with William’s medium containing 10% FBS Fetaclone II, 1% pen/strep, 1% glutamine, 0.023 IU/ml insulin (Sanofi-Aventis, Frankfurt, Germany), 4.7 μg/ml hydrocortisone (Pfizer, Berlin, Germany), 80 μg/ml gentamicin (Ratiopharm, Ulm, Germany) and 1.8% DMSO. Cells were infected with HBV (concentrated virus from HepG2.2.15 supernatant) at an MOI of 200 in the presence of 5% PEG overnight. 10 days post infection, infected HepaRG cells were used as target cells. When effector T cells were added, the medium was replaced to differentiation medium without hydrocortisone. HBeAg in the supernatant was measured with the Enzygnost HBe monoclonal kit (Siemens Healthcare Diagnostics, Eschborn, Germany). Alanine transaminase activity was determined in 32 μl of supernatant using the Reflotron system (Roche Diagnostics). Total DNA was extracted from cells (Macherey & Nagel, Düren, Germany) and cccDNA and rcDNA were quantified as described previously [39]. HBV DNA quantification was done per well and not relative to the cell number, because of unpredictable numbers of proliferating T cells in the total DNA.

### Ethics statement

This study was approved by the local ethical board of the Klinikum rechts der Isar and the University hospital Mainz and written informed consent was obtained from all participants.

## Supporting information

S1 Fig Sensitivity of HBV-specific T-cell clones. 2x10^4^ CD8^+^ T-cell clones specific for peptides C18, S20 or S172 were co-cultured with 2x10^4^ T2 cells pulsed with decreasing peptide concentrations (effector to target ratio, E:T = 1:1) for 4 hours. Green and blue colors indicate clones originating from a donor with resolved infection, clones with yellow and reddish, or grey color originated from acutely infected donors. Cytotoxicity of effector cells was assessed by chromium release assay. Data are presented as mean values +/- SEM from triplicate co-cultures.
(PDF)

S2 Fig Expression of HBV-specific TCRs. (A) Schematic representation of both TCR chains cloned as separate transgene cassette into the retroviral vector MP71. (B-D) CD3 mobilization to the cell surface of Jurkat cells, which do not express an endogenous TCR, indicates expression of a TCR introduced by retroviral transduction. PBMC were pre-gated on CD4^+^ and CD8^+^ T cells. MHC Streptamer and CD3 staining of TCRs two days after retroviral transduction with TCR α and β chains of C18-specific (B), S20-specific (C), or S172-specific (D) T cells. From clone 4G two TCR α chains had been identified and were therefore tested separately in combination with the identified 4G β chain. (E) PBMC were transduced with a pair of retroviruses encoding either TCR α or β chain. Transduced PBMC were co-cultured with T2 cells loaded with 1 μM of peptide (E:T 1:3 up to 1:40, depending on transduction efficiency). After 20 hours, supernatants were analyzed for IFN-γ concentration.
(PDF)

S3 Fig Optimization and expression of HBV-specific TCRs. (A) Strategy for cloning both TCR chains as one transgene cassette into the retroviral vector MP71. To increase TCR expression and pairing after retroviral transduction, gene sequences were codon-optimized, constant regions were murinized with an additional cysteine-bond and TCR α and β chains were fused by a P2A element for polycystronic expression. The variable part of the TCR β chain (TRBV) was synthesized with an overlap to MP71 and the murine constant domain of the β chain (mTRBC) and the variable part of the TCR α chain (TRAV) was synthesized with an overlap to the P2A element and the murine constant domain of the α chain (mTRAC). Both constant domains were amplified by PCR from a TCR template. Variable and constant parts of the respective chains were then annealed and combined in a fusion PCR, followed by a fusion PCR of α and β chain. (B) Exemplary Streptamer staining of PBMC after retroviral transduction with the TCR chains of clone FLP14. Retrovirus supernatant was generated by transfection of 293T cells with virus packaging plasmids and TCR chains on either two separate plasmids (upper panel) or one single plasmid (lower panel). (C) Staining of CD4^+^ T cells transduced with cloned TCRs with an antibody against the murine constant domain of the β chain (mTRBC).
(PDF)

S4 Fig Cross-reactivity of TCR-transduced T cells. 1x10^5^ T2 cells loaded with 1 μM of C18, S20 or S172 were co-cultured with 5x10^5^ T cells (CD8^+^ and CD4^+^) expressing (A) C18-specific, (B) S20-specific, or (C) S172-specific TCRs. IFN-γ and TNF-α single or double positive T cells were detected by intracellular cytokine staining after 5 hours of stimulation at 37°C and overnight rest at 4°C. Data are presented as values from single co-cultures.
(PDF)

S5 Fig Recognition of HBV-negative hepatoma cells by TCR-transduced T cells. Specific lysis of HBV- HepG2 hepatoma cells or T-cell activation (IFN-γ ELISA) by TCR-transduced CD8^+^ (A) or CD4^+^ (B) T cells was measured. After retroviral transduction CD8^+^ and CD4^+^ T cells were separated by MACS. The x-axis indicates the decreasing number of effector cells, which was co-cultured with target cells for 72 hours. HepG2 cells are the parental cell line, from which HBV-replicating cells HepG2.2.15 used in Fig 6 were generated. Each color represents one TCR. Data are presented as mean values +/- SEM from triplicate co-cultures.
(PDF)

S6 Fig Recognition of endogenously processed S172 peptide by T cells transduced with S172-specific TCRs. Specific lysis or IFN-γ secretion of HBV-replicating HepG2.2.15 (A) or HBV^-^ HepG2 (B) hepatoma cells by CD8^+^ or CD4^+^ T cells transduced with S172-specific TCR WL12 (blue) or WL31 (red). After retroviral transduction CD8^+^ and CD4^+^ T cells were separated by MACS. The x-axis indicates the ratio of TCR^+^ effector cells co-cultured with target cells for 72 hours. (C) HeLa cells transduced to stably express HLA-A*02 and transiently transfected with an S-plasmid were co-cultured with two different numbers of T cells. Data are presented as mean values +/- SEM from triplicate co-cultures.
(PDF)

S1 Table Stimulation procedure for isolation of HBV-specific T-cell clones and receptors. (PDF)

S2 Table HBV-specific T-cell receptors. (PDF)

S3 Table Summary of TCR comparison. (PDF)

## References

[1] World Health Organization. Global hepatitis report 2017 Geneva: World Health Organization; 2017. Licence: CC BY-NC-SA 3.0 IGO

[2] Block TM, Gish R, Guo H, Mehta A, Cuconati A, Thomas London W, et al Chronic hepatitis B: what should be the goal for new therapies? Antiviral Research. 2013 4;98(1):27–34. doi: 10.1016/j.antiviral.2013.01.006 23391846 10.1016/j.antiviral.2013.01.006 PMC3627746

[3] Rehermann B. Pathogenesis of chronic viral hepatitis: differential roles of T cells and NK cells. Nature Medicine. 2013 7 8;19(7):859–68. doi: 10.1038/nm.3251 23836236 10.1038/nm.3251 PMC4482132

[4] Thimme R, Wieland S, Steiger C, Ghrayeb J, Reimann KA, Purcell RH, et al CD8+ T Cells Mediate Viral Clearance and Disease Pathogenesis during Acute Hepatitis B Virus Infection. Journal of Virology. 2003 1 1;77(1):68–76. doi: 10.1128/JVI.77.1.68-76.2003 12477811 10.1128/JVI.77.1.68-76.2003 PMC140637

[5] Xia Y, Protzer U. Control of Hepatitis B Virus by Cytokines. Viruses. 2017 1 20;9(1):18. 10.3390/v9010018 PMC5294987 28117695

[6] Bohne F, Protzer U. Adoptive T-cell therapy as a therapeutic option for chronic hepatitis B. Journal of Viral Hepatitis. 2007 11;14(s1):45–50. 17958642 10.1111/j.1365-2893.2007.00913.x

[7] Bohne F, Chmielewski M, Ebert G, Wiegmann K, Kürschner T, Schulze A, et al T Cells Redirected Against Hepatitis B Virus Surface Proteins Eliminate Infected Hepatocytes. Gastroenterology. 2008 1;134(1):239–47. doi: 10.1053/j.gastro.2007.11.002 18166356 10.1053/j.gastro.2007.11.002

[8] Krebs K, Böttinger N, Huang L-R, Chmielewski M, Arzberger S, Gasteiger G, et al T Cells Expressing a Chimeric Antigen Receptor That Binds Hepatitis B Virus Envelope Proteins Control Virus Replication in Mice. Gastroenterology. 2013 8;145(2):456–65. doi: 10.1053/j.gastro.2013.04.047 23639914 10.1053/j.gastro.2013.04.047

[9] Gehring AJ, Xue S-A, Ho ZZ, Teoh D, Ruedl C, Chia A, et al Engineering virus-specific T cells that target HBV infected hepatocytes and hepatocellular carcinoma cell lines. Journal of Hepatology. 2011 7;55(1):103–10. doi: 10.1016/j.jhep.2010.10.025 21145860 10.1016/j.jhep.2010.10.025

[10] Koh S, Shimasaki N, Suwanarusk R, Ho ZZ, Chia A, Banu N, et al A Practical Approach to Immunotherapy of Hepatocellular Carcinoma Using T Cells Redirected Against Hepatitis B Virus. Mol Ther Nucleic Acids. 2013 8;2(8):e114. 10.1038/mtna.2013.43 PMC3759740 23941866

[11] Johnson LA, Morgan RA, Dudley ME, Cassard L, Yang JC, Hughes MS, et al Gene therapy with human and mouse T-cell receptors mediates cancer regression and targets normal tissues expressing cognate antigen. Blood. 2009 7 16;114(3):535–46. doi: 10.1182/blood-2009-03-211714 19451549 10.1182/blood-2009-03-211714 PMC2929689

[12] Morgan RA, Dudley ME, Wunderlich JR, Hughes MS, Yang JC, Sherry RM, et al Cancer Regression in Patients After Transfer of Genetically Engineered Lymphocytes. Science. 2006 10 6;314(5796):126–9. doi: 10.1126/science.1129003 16946036 10.1126/science.1129003 PMC2267026

[13] Pule MA, Savoldo B, Myers GD, Rossig C, Russell HV, Dotti G, et al Virus-specific T cells engineered to coexpress tumor-specific receptors: persistence and antitumor activity in individuals with neuroblastoma. Nature Medicine. 2008 11 2;14(11):1264–70. doi: 10.1038/nm.1882 18978797 10.1038/nm.1882 PMC2749734

[14] Zhong S, Malecek K, Johnson LA, Yu Z, Vega-Saenz de Miera E, Darvishian F, et al T-cell receptor affinity and avidity defines antitumor response and autoimmunity in T-cell immunotherapy. Proceedings of the National Academy of Sciences. 2013 4 23;110(17):6973–8. 10.1073/pnas.1221609110 PMC3637771 23576742

[15] Walker LJ, Sewell AK, Klenerman P. T cell sensitivity and the outcome of viral infection. Clin Exp Immunol. 2010 3;159(3):245–55. doi: 10.1111/j.1365-2249.2009.04047.x 19968665 10.1111/j.1365-2249.2009.04047.x PMC2819491

[16] Sommermeyer D, Hudecek M, Kosasih PL, Gogishvili T, Maloney DG, Turtle CJ, et al Chimeric antigen receptor-modified T cells derived from defined CD8(+) and CD4(+) subsets confer superior antitumor reactivity in vivo. Leukemia. 2016 2;30(2):492–500. doi: 10.1038/leu.2015.247 26369987 10.1038/leu.2015.247 PMC4746098

[17] Muranski P, Restifo NP. Adoptive immunotherapy of cancer using CD4+ T cells. Current Opinion in Immunology. 2009 4;21(2):200–8. doi: 10.1016/j.coi.2009.02.004 19285848 10.1016/j.coi.2009.02.004 PMC2715842

[18] Stone JD, Kranz DM. Role of T Cell Receptor Affinity in the Efficacy and Specificity of Adoptive T Cell Therapies. Front Immunol. Frontiers; 2013;4. 10.3389/fimmu.2013.00244 PMC3748443 23970885

[19] Stone JD, Chervin AS, Kranz DM. T-cell receptor binding affinities and kinetics: impact on T-cell activity and specificity. Immunology. 2009 2;126(2):165–76. doi: 10.1111/j.1365-2567.2008.03015.x 19125887 10.1111/j.1365-2567.2008.03015.x PMC2632691

[20] Desmond CP, Bartholomeusz A, Gaudieri S, Revill PA, Lewin SR. A systematic review of T-cell epitopes in hepatitis B virus: identification, genotypic variation and relevance to antiviral therapeutics. Antivir Ther. 2008;13(2):161–75. 18505168

[21] Nauerth M, Weissbrich B, Knall R, Franz T, Dossinger G, Bet J, et al TCR-Ligand koff Rate Correlates with the Protective Capacity of Antigen-Specific CD8+ T Cells for Adoptive Transfer. Science Translational Medicine. 2013 7 3;5(192):192ra87–7. doi: 10.1126/scitranslmed.3005958 23825303 10.1126/scitranslmed.3005958 PMC3991308

[22] Leisegang M, Engels B, Meyerhuber P, Kieback E, Sommermeyer D, Xue S-A, et al Enhanced functionality of T cell receptor-redirected T cells is defined by the transgene cassette. J Mol Med. 2008 3 12;86(5):573–83. doi: 10.1007/s00109-008-0317-3 18335188 10.1007/s00109-008-0317-3

[23] Loirat D, Lemonnier FA, Michel ML. Multiepitopic HLA-A*0201-restricted immune response against hepatitis B surface antigen after DNA-based immunization. J Immunol. 2000 10 15;165(8):4748–55. 11035120 10.4049/jimmunol.165.8.4748

[24] Urbani S, Boni C, Missale G, Elia G, Cavallo C, Massari M, et al Virus-Specific CD8+ Lymphocytes Share the Same Effector-Memory Phenotype but Exhibit Functional Differences in Acute Hepatitis B and C. Journal of Virology. 2002 12 15;76(24):12423–34. doi: 10.1128/JVI.76.24.12423-12434.2002 12438568 10.1128/JVI.76.24.12423-12434.2002 PMC136708

[25] Busch DH, Pamer EG. T cell affinity maturation by selective expansion during infection. J Exp Med. 1999 2 15;189(4):701–10. 9989985 10.1084/jem.189.4.701 PMC2192934

[26] Venturi V, Price DA, Douek DC, Davenport MP. The molecular basis for public T-cell responses? Nat Rev Immunol. 2008 3;8(3):231–8. doi: 10.1038/nri2260 18301425 10.1038/nri2260

[27] Yang J, Chen J, He J, Xie Y, Zhu Y, Cao H, et al Profiling the repertoire of T-cell receptor beta-chain variable genes in peripheral blood lymphocytes from subjects who have recovered from acute hepatitis B virus infection. Cell Mol Immunol. 2014 7;11(4):332–42. doi: 10.1038/cmi.2014.22 25126662 10.1038/cmi.2014.22 PMC4085520

[28] Marrack P, Scott-Browne JP, Dai S, Gapin L, Kappler JW. Evolutionarily Conserved Amino Acids That Control TCR-MHC Interaction. Annu Rev Immunol. 2008 4;26(1):171–203. 18304006 10.1146/annurev.immunol.26.021607.090421 PMC3164820

[29] Cohen CJ, Zhao Y, Zheng Z, Rosenberg SA, Morgan RA. Enhanced antitumor activity of murine-human hybrid T-cell receptor (TCR) in human lymphocytes is associated with improved pairing and TCR/CD3 stability. Cancer Research. 2006 9 1;66(17):8878–86. doi: 10.1158/0008-5472.CAN-06-1450 16951205 10.1158/0008-5472.CAN-06-1450 PMC2147082

[30] Reuß S, Sebestyén Z, Heinz N, Loew R, Baum C, Debets R, et al TCR-engineered T cells: A model of inducible TCR expression to dissect the interrelationship between two TCRs. Eur J Immunol. 2013 10 20;44(1):265–74. doi: 10.1002/eji.201343591 24114521 10.1002/eji.201343591 PMC4209802

[31] Bendle GM, Linnemann C, Hooijkaas AI, Bies L, de Witte MA, Jorritsma A, et al Lethal graft-versus-host disease in mouse models of T cell receptor gene therapy. Nature Medicine. 2010 4 18;16(5):565–70. doi: 10.1038/nm.2128 20400962 10.1038/nm.2128

[32] Derby M, Alexander-Miller M, Tse R, Berzofsky J. High-avidity CTL exploit two complementary mechanisms to provide better protection against viral infection than low-avidity CTL. J Immunol. 2001 2 1;166(3):1690–7. 11160212 10.4049/jimmunol.166.3.1690

[33] Tan AT, Loggi E, Boni C, Chia A, Gehring AJ, Sastry KSR, et al Host Ethnicity and Virus Genotype Shape the Hepatitis B Virus-Specific T-Cell Repertoire. Journal of Virology. 2008 10 24;82(22):10986–97. doi: 10.1128/JVI.01124-08 18799575 10.1128/JVI.01124-08 PMC2573267

[34] Webster GJ, Reignat S, Maini MK, Whalley SA, Ogg GS, King A, et al Incubation phase of acute hepatitis B in man: dynamic of cellular immune mechanisms. Hepatology. 2000 11;32(5):1117–24. doi: 10.1053/jhep.2000.19324 11050064 10.1053/jhep.2000.19324

[35] Maini MK, Boni C, Ogg GS, King AS, Reignat S, Lee CK, et al Direct ex vivo analysis of hepatitis B virus-specific CD8(+) T cells associated with the control of infection. Gastroenterology. 1999 12;117(6):1386–96. 10579980 10.1016/s0016-5085(99)70289-1

[36] Bertoni R, Sidney J, Fowler P, Chesnut RW, Chisari FV, Sette A. Human histocompatibility leukocyte antigen-binding supermotifs predict broadly cross-reactive cytotoxic T lymphocyte responses in patients with acute hepatitis. J Clin Invest. American Society for Clinical Investigation; 1997 8 1;100(3):503–13. doi: 10.1172/JCI119559 9239396 10.1172/JCI119559 PMC508216

[37] Engels B, Engelhard VH, Sidney J, Sette A, Binder DC, Liu RB, et al Relapse or Eradication of Cancer Is Predicted by Peptide-Major Histocompatibility Complex Affinity. Cancer Cell. 2013 4;23(4):516–26. doi: 10.1016/j.ccr.2013.03.018 23597565 10.1016/j.ccr.2013.03.018 PMC3658176

[38] Virgin HW, Wherry EJ, Ahmed R. Redefining Chronic Viral Infection. Cell. 2009 7;138(1):30–50. doi: 10.1016/j.cell.2009.06.036 19596234 10.1016/j.cell.2009.06.036

[39] Lucifora J, Xia Y, Reisinger F, Zhang K, Stadler D, Cheng X, et al Specific and Nonhepatotoxic Degradation of Nuclear Hepatitis B Virus cccDNA. Science. 2014 3 13;343(6176):1221–8. doi: 10.1126/science.1243462 24557838 10.1126/science.1243462 PMC6309542

[40] Xia Y, Stadler D, Lucifora J, Reisinger F, Webb D, Hösel M, et al Interferon-γ and Tumor Necrosis Factor-α Produced by T Cells Reduce the HBV Persistence Form, cccDNA, Without Cytolysis. Gastroenterology. 2016 1;150(1):194–205. doi: 10.1053/j.gastro.2015.09.026 26416327 10.1053/j.gastro.2015.09.026

[41] Hoh A, Heeg M, Ni Y, Schuch A, Binder B, Hennecke N, et al Hepatitis B Virus-Infected HepG2hNTCP Cells Serve as a Novel Immunological Tool To Analyze the Antiviral Efficacy of CD8+ T Cells In Vitro. Journal of Virology. 2015 7;89(14):7433–8. doi: 10.1128/JVI.00605-15 25972537 10.1128/JVI.00605-15 PMC4473563

[42] Sommermeyer D, Conrad H, Krönig H, Gelfort H, Bernhard H, Uckert W. NY-ESO-1 antigen-reactive T cell receptors exhibit diverse therapeutic capability. Int J Cancer. 2013 3 15;132(6):1360–7. doi: 10.1002/ijc.27792 22907642 10.1002/ijc.27792 PMC3617456

[43] Hombrink P, Raz Y, Kester MGD, Boer R, Weißbrich B, dem Borne PA, et al Mixed functional characteristics correlating with TCR‐ligand koff‐rate of MHC‐tetramer reactive T cells within the naive T‐cell repertoire. Eur J Immunol. 2013 11 1;43(11):3038–50. doi: 10.1002/eji.201343397 23893393 10.1002/eji.201343397

[44] Corse E, Gottschalk RA, Allison JP. Strength of TCR-Peptide/MHC Interactions and In Vivo T Cell Responses. The Journal of Immunology. 2011 4 19;186(9):5039–45. doi: 10.4049/jimmunol.1003650 21505216 10.4049/jimmunol.1003650

[45] Liu J, Chen KY, Ren EC. Structural insights into the binding of hepatitis B virus core peptide to HLA-A2 alleles: Towards designing better vaccines. Eur J Immunol. 2011 6 27;41(7):2097–106. doi: 10.1002/eji.201041370 21538979 10.1002/eji.201041370

[46] Engels B, Cam H, Schüler T, Indraccolo S, Gladow M, Baum C, et al Retroviral Vectors for High-Level Transgene Expression in T Lymphocytes. Human Gene Therapy. 2003 8 10;14(12):1155–68. doi: 10.1089/104303403322167993 12908967 10.1089/104303403322167993

[47] Sommermeyer D, Uckert W. Minimal Amino Acid Exchange in Human TCR Constant Regions Fosters Improved Function of TCR Gene-Modified T Cells. The Journal of Immunology. 2010 5 18;184(11):6223–31. doi: 10.4049/jimmunol.0902055 20483785 10.4049/jimmunol.0902055

