# Supplementary material for: Isolation and functional characterization of hepatitis B virus-specific T-cell receptors as new tools for experimental and clinical use
Source: PLoS One. 2017 Aug 8;12(8):e0182936. doi: 10.1371/journal.pone.0182936 (PMC5549754; doi:10.1371/journal.pone.0182936)
Supplement: S1 Fig — 2x10^4^ CD8+ T-cell clones specific for peptides C18, S20 or S172 were co-cultured with 2x10^4^ T2 cells pulsed with decreasing peptide concentrations (effector to target ratio, E:T = 1:1) for 4 hours. Green and blue colors indicate clones originating from a donor with resolved infection, clones with yellow and reddish, or grey color originated from acutely infected donors. Cytotoxicity of effector cells was assessed by chromium release assay. Data are presented as mean values +/- SEM from triplicate co-cultures. (PDF) [file pone.0182936.s001.pdf]

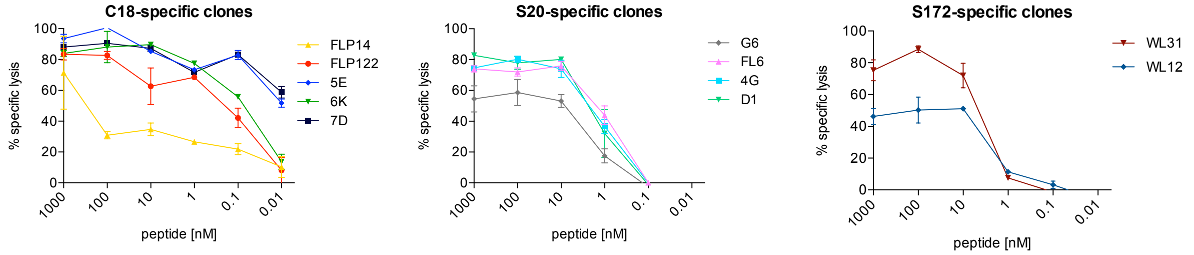

S1 Fig. Sensitivity of HBV-specific T-cell clones. 2x10^4 $CD8^+$ T cell clones specific for peptides C18, S20 or S172 were co-cultured with 2x10^4 T2 cells pulsed with decreasing peptide concentrations (effector to target ratio, E:T = 1:1) for 4 hours. Green and blue colors indicate clones originating from a donor with resolved infection, clones with yellow and reddish, or grey color originated from acutely infected donors. Cytotoxicity of effector cells was assessed by chromium release assay. Data are presented as mean values +/- SEM from triplicate co-cultures.
