# Supplementary material for: Isolation and functional characterization of hepatitis B virus-specific T-cell receptors as new tools for experimental and clinical use
Source: PLoS One. 2017 Aug 8;12(8):e0182936. doi: 10.1371/journal.pone.0182936 (PMC5549754; doi:10.1371/journal.pone.0182936)
Supplement: S2 Fig — (A) Schematic representation of both TCR chains cloned as separate transgene cassette into the retroviral vector MP71. (B-D) CD3 mobilization to the cell surface of Jurkat cells, which do not express an endogenous TCR, indicates expression of a TCR introduced by retroviral transduction. PBMC were pre-gated on CD4+ and CD8+ T cells. MHC Streptamer and CD3 staining of TCRs two days after retroviral transduction with TCR α and β chains of C18-specific (B), S20-specific (C), or S172-specific (D) T cells. From clone 4G two TCR α chains had been identified and were therefore tested separately in combination with the identified 4G β chain. (E) PBMC were transduced with a pair of retroviruses encoding either TCR α or β chain. Transduced PBMC were co-cultured with T2 cells loaded with 1 μM of peptide (E:T 1:3 up to 1:40, depending on transduction efficiency). After 20 hours, supernatants were analyzed for IFN-γ concentration. (PDF) [file pone.0182936.s002.pdf]

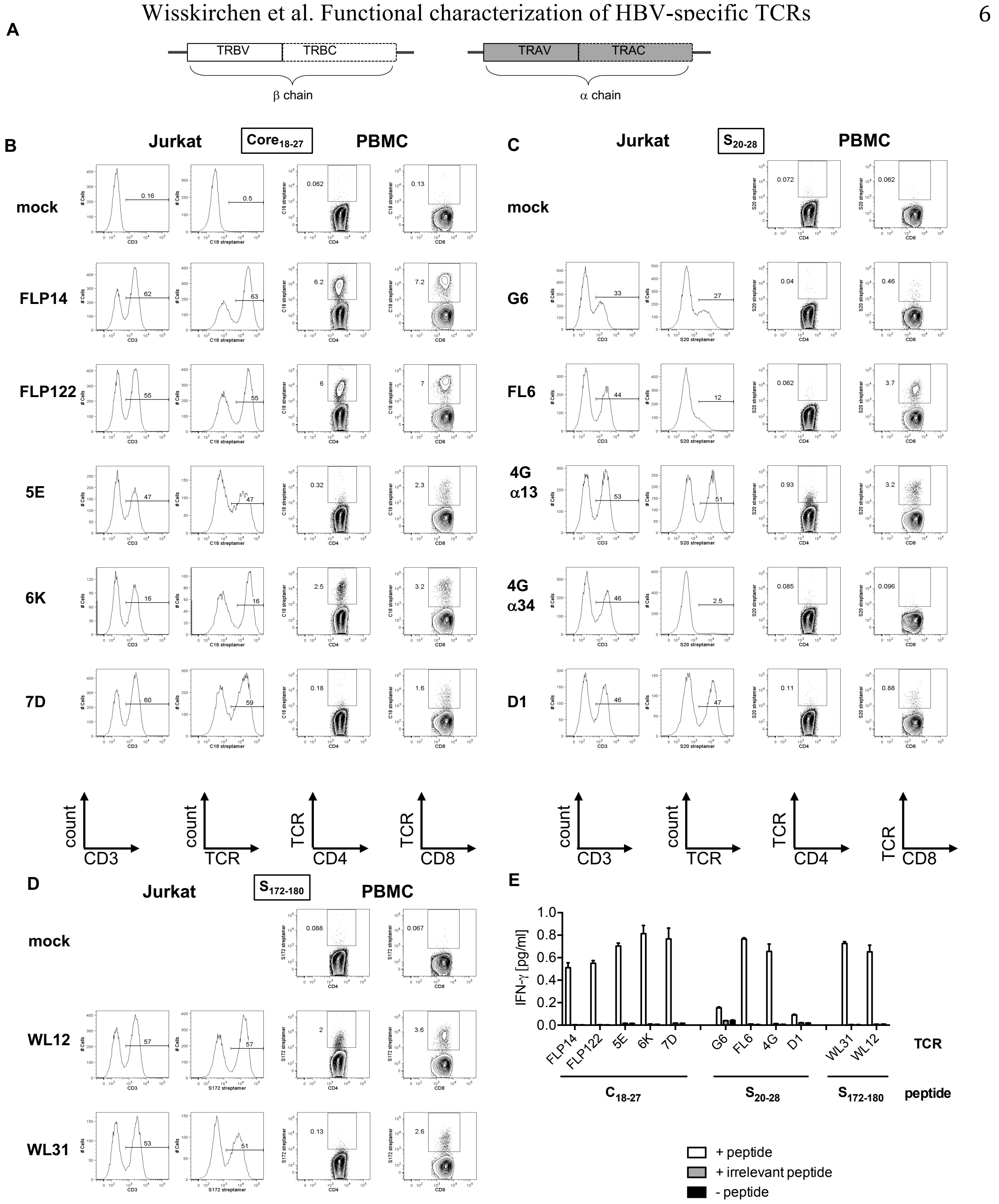

S2 Fig. Expression of HBV-specific TCRs. (A) Schematic representation of both TCR chains cloned as separate transgene cassette into the retroviral vector MP71. (B-D) CD3 mobilization to the cell surface of Jurkat cells that do not express an endogenous TCR indicates expression of a TCR introduced by retroviral transduction. PBMC were pre-gated on $CD4^{+}$ and $CD8^{+}$ T cells. MHC Streptamer and CD3 staining of TCRs two days after retroviral transduction with TCR α and β chains of C18-specific (B), S20-specific (C), or S172-specific (D) T cells. From clone 4G two TCR α chains had been identified and were therefore tested separately in combination with the identified 4G β chain. (E) PBMC were transduced with a pair of retroviruses encoding either TCR α or β chain. Transduced PBMC were co-cultured with T2 cells loaded with 1 μM of peptide (E:T 1:3 up to 1:40, depending on transduction efficiency). After 20 hours, supernatants were analyzed for IFN-γ concentration.
