# Supplementary material for: Isolation and functional characterization of hepatitis B virus-specific T-cell receptors as new tools for experimental and clinical use
Source: PLoS One. 2017 Aug 8;12(8):e0182936. doi: 10.1371/journal.pone.0182936 (PMC5549754; doi:10.1371/journal.pone.0182936)
Supplement: S3 Fig — (A) Strategy for cloning both TCR chains as one transgene cassette into the retroviral vector MP71. To increase TCR expression and pairing after retroviral transduction, gene sequences were codon-optimized, constant regions were murinized with an additional cysteine-bond and TCR α and β chains were fused by a P2A element for polycystronic expression. The variable part of the TCR β chain (TRBV) was synthesized with an overlap to MP71 and the murine constant domain of the β chain (mTRBC) and the variable part of the TCR α chain (TRAV) was synthesized with an overlap to the P2A element and the murine constant domain of the α chain (mTRAC). Both constant domains were amplified by PCR from a TCR template. Variable and constant parts of the respective chains were then annealed and combined in a fusion PCR, followed by a fusion PCR of α and β chain. (B) Exemplary Streptamer staining of PBMC after retroviral transduction with the TCR chains of clone FLP14. Retrovirus supernatant was generated by transfection of 293T cells with virus packaging plasmids and TCR chains on either two separate plasmids (upper panel) or one single plasmid (lower panel). (C) Staining of CD4+ T cells transduced with cloned TCRs with an antibody against the murine constant domain of the β chain (mTRBC). (PDF) [file pone.0182936.s003.pdf]

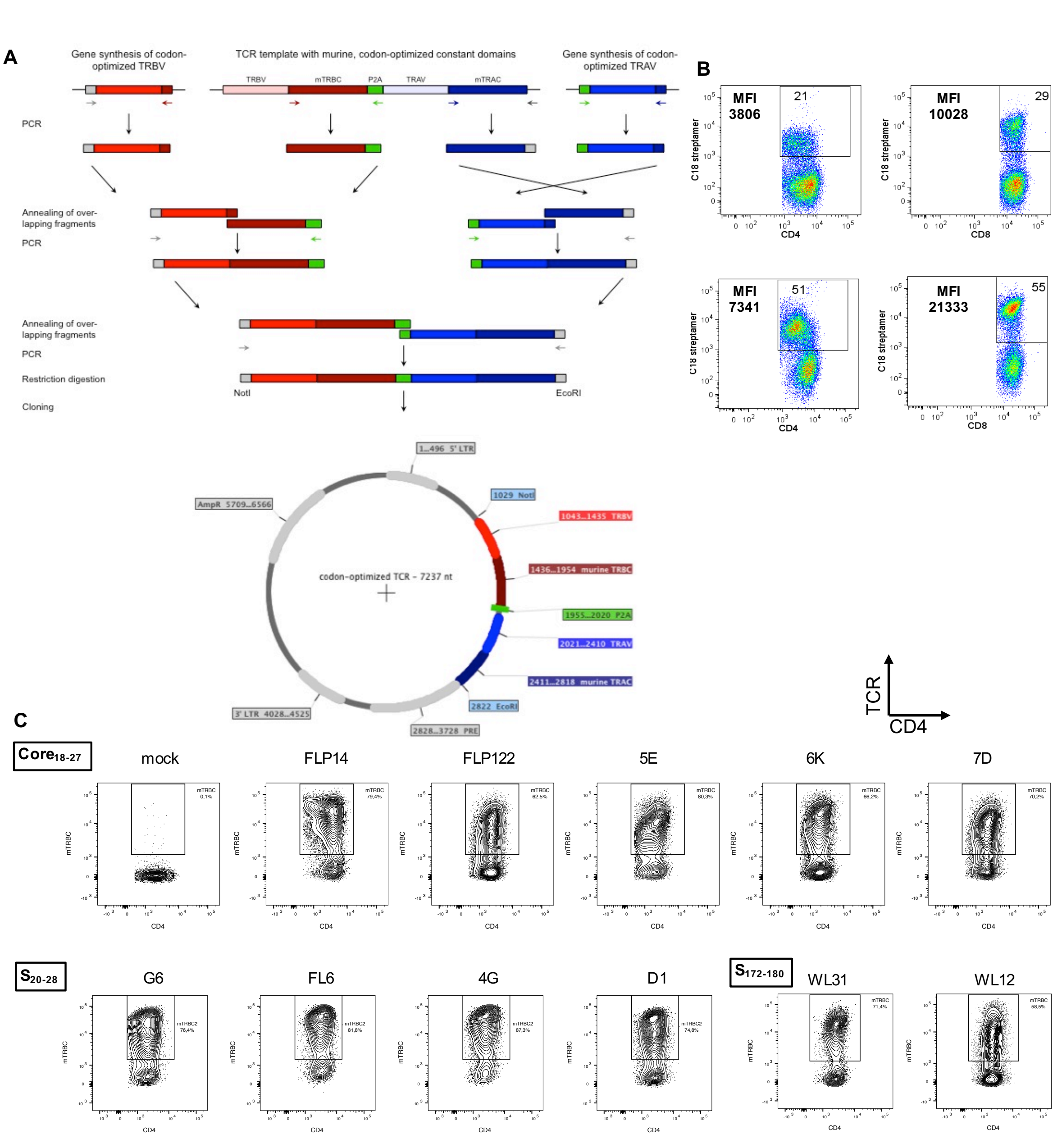

S3 Fig. Optimization and expression of HBV-specific TCRs. (A) Strategy for cloning both TCR chains as one transgene cassette into the retroviral vector MP71. To increase TCR expression and pairing after retroviral transduction, gene sequences were codon-optimized, constant regions were murinized with an additional cysteine-bond and TCR α and β chains were fused by a P2A element for polycystronic expression. The variable part of the TCR β chain (TRBV) was synthesized with an overlap to MP71 and the murine constant domain of the β chain (mTRBC) and the variable part of the TCR α chain (TRAV) was synthesized with an overlap to the P2A element and the murine constant domain of the α chain (mTRAC). Both constant domains were amplified by PCR from a TCR template. Variable and constant parts of the respective chains were then annealed and combined in a fusion PCR, followed by a fusion PCR of α and β chain. (B) Exemplary streptamer staining of PBMC after retroviral transduction with the TCR chains of clone FLP14. Retrovirus supernatant was generated by transfection of 293T cells with virus packaging plasmids and TCR chains on either two separate plasmids (upper panel) or one single plasmid (lower panel). (C) Staining of $CD4^+$ T cells grafted with cloned TCRs with an antibody against the murine constant domain of the β chain (mTRBC).
