# Supplementary material for: Isolation and functional characterization of hepatitis B virus-specific T-cell receptors as new tools for experimental and clinical use
Source: PLoS One. 2017 Aug 8;12(8):e0182936. doi: 10.1371/journal.pone.0182936 (PMC5549754; doi:10.1371/journal.pone.0182936)
Supplement: S4 Fig — 1x10^5^ T2 cells loaded with 1 μM of C18, S20 or S172 were co-cultured with 5x10^5^ T cells (CD8+ and CD4+) expressing (A) C18-specific, (B) S20-specific, or (C) S172-specific TCRs. IFN-γ and TNF-α single or double positive T cells were detected by intracellular cytokine staining after 5 hours of stimulation at 37°C and overnight rest at 4°C. Data are presented as values from single co-cultures. (PDF) [file pone.0182936.s004.pdf]

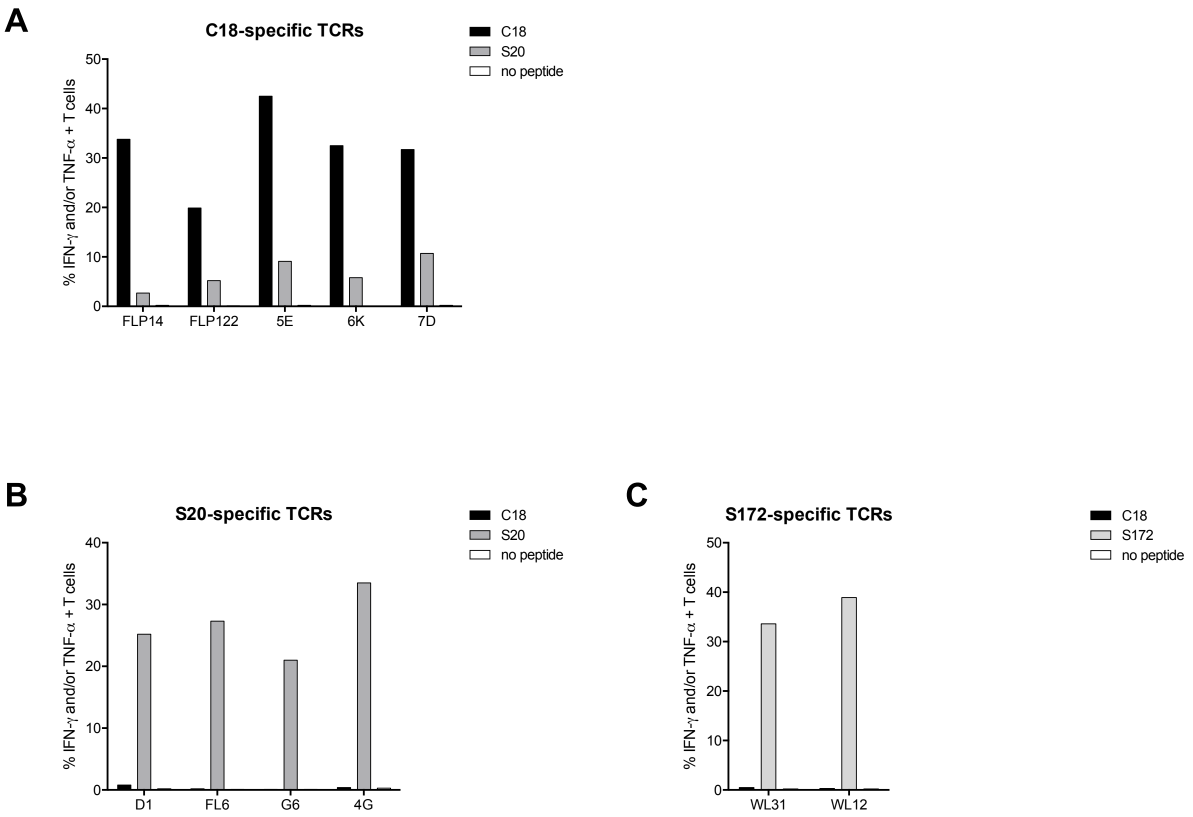

S4 Fig. Cross-reactivity of TCR-transduced T cells.

1x10^5 T2 cells loaded with 1μM of C18, S20 or S172 were co-cultured with 5x10^5 T cells ($CD8^+$ and $CD4^+$) expressing (A) C18-specific, (B) S20-specific, or (C) S172-specific TCRs. IFN-γ and TNF-α single or double positive T cells were detected by intracellular cytokine staining after 5 hours of stimulation at 37°C and overnight rest at 4°C. Data are presented as values from single co-cultures.
