# Supplementary material for: Isolation and functional characterization of hepatitis B virus-specific T-cell receptors as new tools for experimental and clinical use
Source: PLoS One. 2017 Aug 8;12(8):e0182936. doi: 10.1371/journal.pone.0182936 (PMC5549754; doi:10.1371/journal.pone.0182936)
Supplement: S5 Fig — Specific lysis of HBV- HepG2 hepatoma cells or T-cell activation (IFN-γ ELISA) by TCR-transduced CD8+ (A) or CD4+ (B) T cells was measured. After retroviral transduction CD8+ and CD4+ T cells were separated by MACS. The x-axis indicates the decreasing number of effector cells, which was co-cultured with target cells for 72 hours. HepG2 cells are the parental cell line, from which HBV-replicating cells HepG2.2.15 used in Fig 6 were generated. Each color represents one TCR. Data are presented as mean values +/- SEM from triplicate co-cultures. (PDF) [file pone.0182936.s005.pdf]

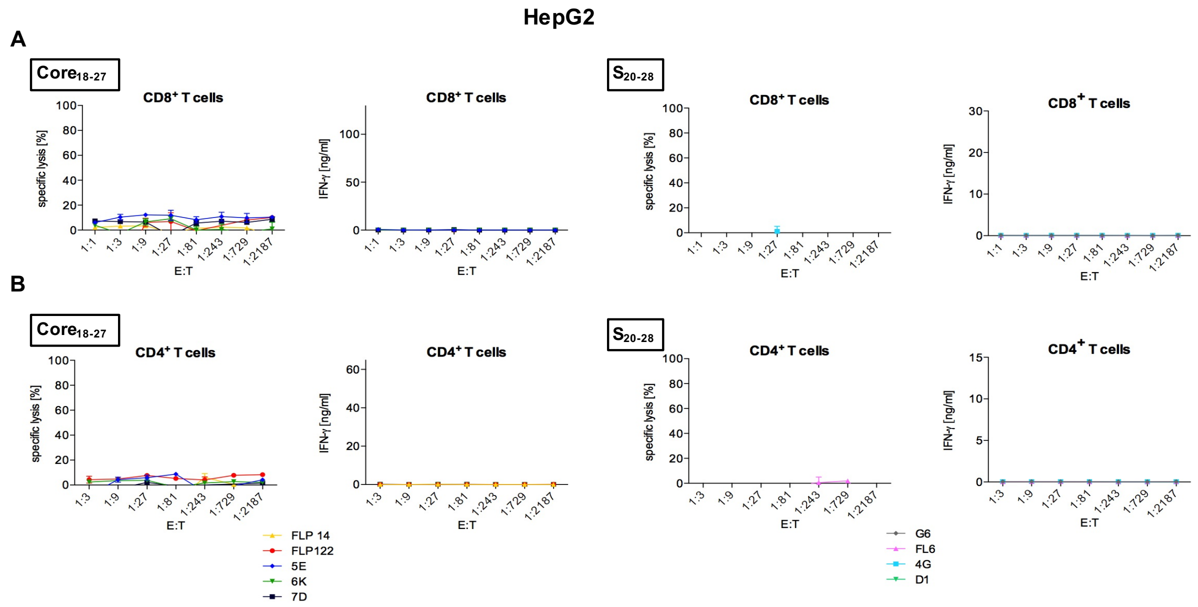

S5 Fig. Recognition of HBV negative hepatoma cells by TCR-grafted T cells.

Specific lysis of HBV- HepG2 hepatoma cells or T-cell activation (IFN-γ ELISA) by TCR-grafted CD8$^+$ (A) or CD4$^+$ (B) T cells was measured. After retroviral transduction CD8$^+$ and CD4$^+$ T cells were separated by MACS. The x-axis indicates the decreasing number of effector cells, which was co-cultured with target cells for 72 hours. HepG2 cells are the parental cell line, from which HBV-replicating cells HepG2.2.15 used in Fig 6 were generated. Each color represents one TCR. Data are presented as mean values +/- SEM from triplicate co-cultures.
