# Supplementary material for: Isolation and functional characterization of hepatitis B virus-specific T-cell receptors as new tools for experimental and clinical use
Source: PLoS One. 2017 Aug 8;12(8):e0182936. doi: 10.1371/journal.pone.0182936 (PMC5549754; doi:10.1371/journal.pone.0182936)
Supplement: S6 Fig — Specific lysis or IFN-γ secretion of HBV-replicating HepG2.2.15 (A) or HBV- HepG2 (B) hepatoma cells by CD8+ or CD4+ T cells transduced with S172-specific TCR WL12 (blue) or WL31 (red). After retroviral transduction CD8+ and CD4+ T cells were separated by MACS. The x-axis indicates the ratio of TCR+ effector cells co-cultured with target cells for 72 hours. (C) HeLa cells transduced to stably express HLA-A*02 and transiently transfected with an S-plasmid were co-cultured with two different numbers of T cells. Data are presented as mean values +/- SEM from triplicate co-cultures. (PDF) [file pone.0182936.s006.pdf]

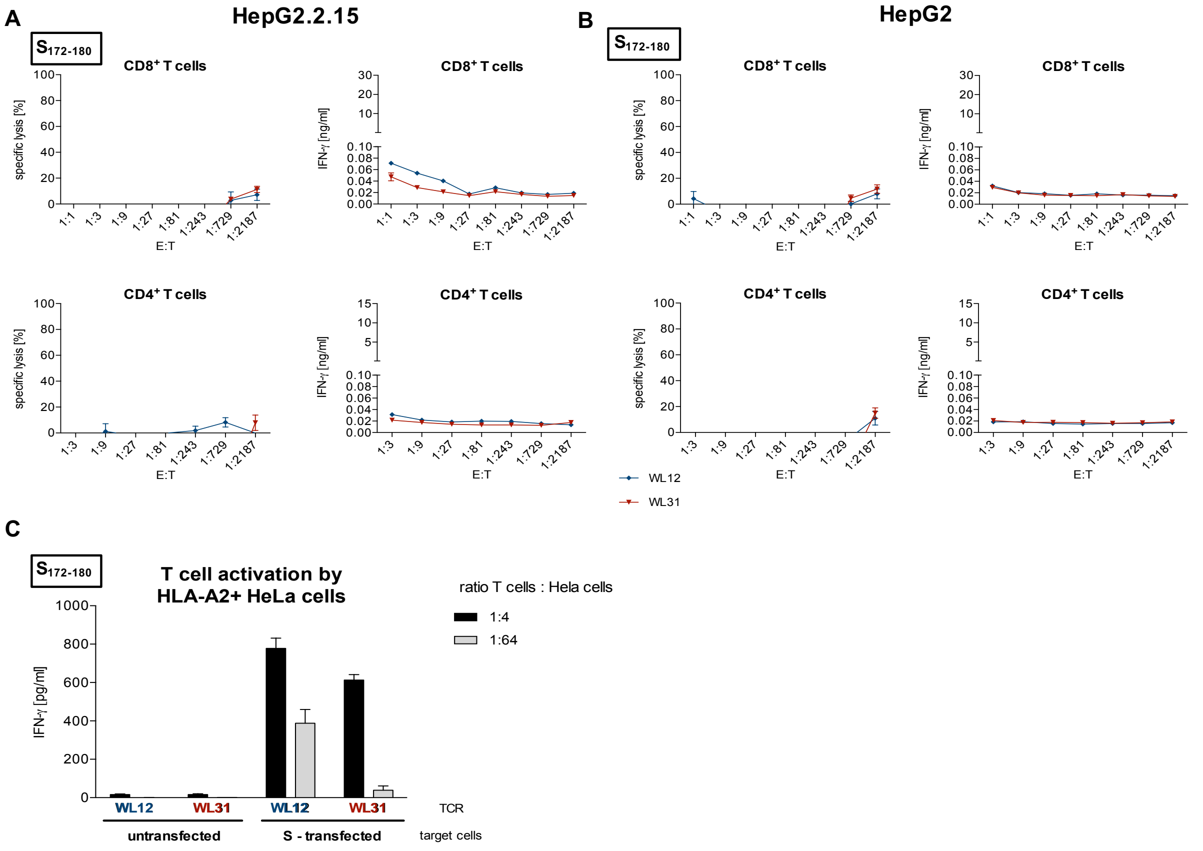

S6 Fig. Recognition of endogenously processed S172 peptide by T cells grafted with S172-specific TCRs.

Specific lysis or IFN-γ secretion of HBV-replicating HepG2.2.15 (A) or HBV^{-} HepG2 (B) hepatoma cells by CD8^{+} or CD4^{+} T cells grafted with S172-specific TCR WL12 (blue) or WL31 (red). After retroviral transduction CD8^{+} and CD4^{+} T cells were separated by MACS. The x-axis indicates the ratio of TCR^{+} effector cells co-cultured with target cells for 72 hours. (C) HeLa cells transduced to stably express HLA-A*02 and transiently transfected with an S-plasmid were co-cultured with two different numbers of T cells. Data are presented as mean values +/- SEM from triplicate co-cultures.
