# Supplementary material for: Isolation and functional characterization of hepatitis B virus-specific T-cell receptors as new tools for experimental and clinical use
Source: PLoS One. 2017 Aug 8;12(8):e0182936. doi: 10.1371/journal.pone.0182936 (PMC5549754; doi:10.1371/journal.pone.0182936)
Supplement: S1 Table — (PDF) [file pone.0182936.s007.pdF]

TCR	Donor	Hepatitis B status	Specificity	1. Stimulation		2. Stimulation	
				Peptide [ ]	APC : Donor cells	Peptide [ ]	APC : Donor cells
G6	3	acute	S20	0,8 nM	- : 9x10^{5} PBMC	1µM	d14, 1x10^{4} T2 : ≈2x10^{6} PBMC
FL6	2	acute	S20	1nM	1x10^{5} T2 : 2x10^{6} PBMC	100nM	d7, 1x10^{5} T2 : ≈2x10^{6} PBMC
4G	1	resolved	S20	1nM	1x10^{5} T2: 1,1x10^{6} PBMC	-	-
D1	1	resolved	S20	1nM	1x10^{5} T2: 1,1x10^{6} PBMC	1µM	d14, 1x10^{4} T2 : ≈2x10^{6} PBMC
WL31	2	acute	S172	1nM	1x10^{5} T2 : 2x10^{6} PBMC	100nM	d7, 1x10^{5} T2 : ≈2x10^{6} PBMC
WL12	1	resolved	S172	1nM	1x10^{5} T2: 1x10^{6} CD8^{+}	100nM	d7, 1x10^{5} T2 : ≈2x10^{6} CD8^{+}
FLP14	2	acute	C18	1nM	1x10^{5} T2 : 2x10^{6} PBMC	100nM	d7, 1x10^{5} T2 : ≈2x10^{6} PBMC
FLP122	2	acute	C18	1nM	1x10^{5} T2 : 2x10^{6} PBMC	100nM	d7, 1x10^{5} T2 : ≈2x10^{6} PBMC
5E	1	resolved	C18	1µM	- : 1x10^{6} PBMC	-	-
6K	1	resolved	C18	1µM	1x10^{5} T2: 1,1x10^{6} PBMC	-	-
7D	1	resolved	C18	1nM	1x10^{5} T2: 1,1x10^{6} PBMC	-	-

S1 Table. Stimulation procedure for isolation of HBV-specific T-cell clones and receptors. This table indicates the cell numbers, cell types and peptide concentration that were used to stimulate growth of HBV-specific T-cell clones.
