# Supplementary material for: Isolation and functional characterization of hepatitis B virus-specific T-cell receptors as new tools for experimental and clinical use
Source: PLoS One. 2017 Aug 8;12(8):e0182936. doi: 10.1371/journal.pone.0182936 (PMC5549754; doi:10.1371/journal.pone.0182936)
Supplement: S2 Table — (PDF) [file pone.0182936.s008.pdF]

TCR	Donor	Hepatitis B status	Specificity	TCR chains	
				Vα	Vβ
G6	3	acute	S20	17	12-3
FL6	2	acute	S20	22	12-3
4G	1	resolved	S20	34	5-1
D1	1	resolved	S20	17	12-3
WL31	2	acute	S172	39	6-5
WL12	1	resolved	S172	12-2	7-6
FLP14	2	acute	C18	12-2	2
FLP122	2	acute	C18	12-2	27
5E	1	resolved	C18	13-1	13
6K	1	resolved	C18	13-1	27
7D	1	resolved	C18	17	24-1

S2 Table. TCR chain usage of isolated HBV-specific TCRs. This table indicates, which TCR chains were identified by sequencing and blasting against the IMGT database for the individual T-cell clones.
