# Supplementary material for: Isolation and functional characterization of hepatitis B virus-specific T-cell receptors as new tools for experimental and clinical use
Source: PLoS One. 2017 Aug 8;12(8):e0182936. doi: 10.1371/journal.pone.0182936 (PMC5549754; doi:10.1371/journal.pone.0182936)
Supplement: S3 Table — (PDF) [file pone.0182936.s009.pdF]

	TCR	FLP14	FLP122	5E	6K	7D	G6	FL6	4G	D1	WL31	WL12
	Specificity	$Core_{18-27}$	$Core_{18-27}$	$Core_{18-27}$	$Core_{18-27}$	$Core_{18-27}$	S_{20-28}	S_{20-28}	S_{20-28}	S_{20-28}	$S_{172-180}$	$S_{172-180}$
	Donor	2	2	1	1	1	3	2	1	2	2	1
	Status	acute	acute	resolved	resolved	resolved	acute	acute	resolved	acute	acute	resolved
Cell type	Analysis											
CD8	% Streptamer+	79,10	70,30	82,90	67,60	72,90	68,10	73,80	85,30	72,80	73,40	88,10
CD8	1. EC_{50} peptide [nM] specific lysis	0,05	0,20	0,04	0,06	0,04	1,69	0,57	0,34	0,75	1,34	0,70
CD8	2. peptide [nM] cytokine secretion	0,005	0,004	0,003	0,019	0,005	0,117	0,038	0,032	0,026	4,370	10,200
CD8	3. $E{:}T_{50}$ specific lysis	1:14	1:10	1:20	1:12	1:18	1:4	1:8	1:16	1:19	n.d.	n.d.
CD8	4. E:T IFN-γ secretion	1:215	1:80	1:158	1:290	1:81	1:22	1:45	1:56	1:26	n.d.	n.d.
CD4	% Streptamer+	68,70	39,60	40,00	54,10	16,00	26,00	3,20	70,00	43,40	2,52	64,20
CD4	1. EC_{50} peptide [nM] specific lysis	0,68	n.d.	0,23	0,12	0,49	2924,00	25,07	0,79	4,07	7264,00	1,26
CD4	2. peptide [nM] cytokine secretion	0,40	n.d.	0,08	0,01	0,07	108,30	0,58	0,11	3,16	n.d.	0,47
CD4	3. $E{:}T_{50}$ specific lysis	n.d.	n.d.	1:21	1:19	1:8	n.d.	n.d.	n.d.	n.d.	n.d.	n.d.
CD4	4. E:T IFN-γ secretion	n.d.	n.d.	1:12	1:124	1:5	n.d.	n.d.	5,2	n.d.	n.d.	n.d.
CD3	# of HLA-A*02 subtypes recognized	10	8	10	7	8	5	4	8	5	8	7
	Score	20	13	32	33	26	12	18	31	20	12	18

color code:	Score
superior	4
good	3
intermediate	2
bad	1
n.d.= not detected	0

Streptamer staining was not included in the scoring, because it does not have any implication for using a TCR in T cell therapy

S3 Table. Summary of TCR comparison. This table summarizes the actual values that were measured for each TCR in the different assays for comparing the functionality of the T-cell receptors. For each assay there was a score, grading the performance of a TCR compared to other TCRs. The lower the peptide concentration or the E:T ratio needed to achieve specific lysis or cytokine secretion, the more sensitive is the respective TCR. Assay 1: Peptide concentration needed to kill 50% of peptide-loaded T2 target cells, measured by chromium release assay (see Fig 4). Assay 2: Peptide concentration needed to induce cytokine secretion in at least 10% of TCR^+ T cells, measured by ICS (see Fig 3). Assay 3: E:T ratio needed to kill 50% of HBV^+ hepatoma cells, measured by XTT assay (see Fig 6). Assay 4: E:T ratio needed to induce secretion of 5 ng/ml of IFN-γ measured by ELISA (see Fig 6).
